# Knockdown of NEAT1 prevents post-stroke lipid droplet agglomeration in microglia by regulating autophagy

**DOI:** 10.1007/s00018-023-05045-7

**Published:** 2024-01-12

**Authors:** Yongli Pan, Wenqiang Xin, Wei Wei, Lars Tatenhorst, Irina Graf, Aurel Popa-Wagner, Stefan T. Gerner, Sabine E. Huber, Ertugrul Kilic, Dirk M. Hermann, Mathias Bähr, Hagen B. Huttner, Thorsten R. Doeppner

**Affiliations:** 1 Department of Neurology, University Medical Center Göttingen, Göttingen, Germany; 2 Department of Neurology, University Hospital Essen, University of Duisburg-Essen, Essen, Germany; 3 Department of Neurology, University of Giessen Medical School, Giessen, Germany; 4 Department of Physiology, Faculty of Medicine, Istanbul Medeniyet University, Istanbul, Turkey; 5 Department of Anatomy and Cell Biology, Medical University of Varna, Varna, Bulgaria; 6 Center for Mind, Brain and Behavior (CMBB), University of Marburg and Justus Liebig University Giessen, Giessen, Germany; 7 Research Institute for Health Sciences and Technologies (SABITA), Medipol University, Istanbul, Turkey

**Keywords:** Autophagy, Lipid droplets, Microglia, NEAT1, Stroke

## Abstract

**Background:**

Lipid droplets (LD), lipid-storing organelles containing neutral lipids like glycerolipids and cholesterol, are increasingly accepted as hallmarks of inflammation. The nuclear paraspeckle assembly transcript 1 (NEAT1), a long non-coding RNA with over 200 nucleotides, exerts an indispensable impact on regulating both LD agglomeration and autophagy in multiple neurological disorders. However, knowledge as to how NEAT1 modulates the formation of LD and associated signaling pathways is limited.

**Methods:**

In this study, primary microglia were isolated from newborn mice and exposed to oxygen-glucose-deprivation/reoxygenation (OGD/R). To further explore NEAT1-dependent mechanisms, an antisense oligonucleotide (ASO) was adopted to silence NEAT1 under in vitro conditions. Studying NEAT1-dependent interactions with regard to autophagy and LD agglomeration under hypoxic conditions, the inhibitor and activator of autophagy 3-methyladenine (3-MA) and rapamycin (RAPA) were used, respectively. In a preclinical stroke model, mice received intraventricular injections of ASO NEAT1 or control vectors in order to yield NEAT1 knockdown. Analysis of readout parameters included qRT-PCR, immunofluorescence, western blot assays, and behavioral tests.

**Results:**

Microglia exposed to OGD/R displayed a temporal pattern of NEAT1 expression, peaking at four hours of hypoxia followed by six hours of reoxygenation. After effectively silencing NEAT1, LD formation and autophagy-related proteins were significantly repressed in hypoxic microglia. Stimulating autophagy in ASO NEAT1 microglia under OGD/R conditions by means of RAPA reversed the downregulation of LD agglomeration and perilipin 2 (PLIN2) expression. On the contrary, application of 3-MA promoted repression of both LD agglomeration and expression of the LD-associated protein PLIN2. Under in vivo conditions, NEAT1 was significantly increased in mice at 24 h post-stroke. Knockdown of NEAT1 significantly alleviated LD agglomeration and inhibited autophagy, resulting in improved cerebral perfusion, reduced brain injury and increased neurological recovery.

**Conclusion:**

NEAT1 is a key player of LD agglomeration and autophagy stimulation, and NEAT1 knockdown provides a promising therapeutic value against stroke.

**Graphical abstract:**

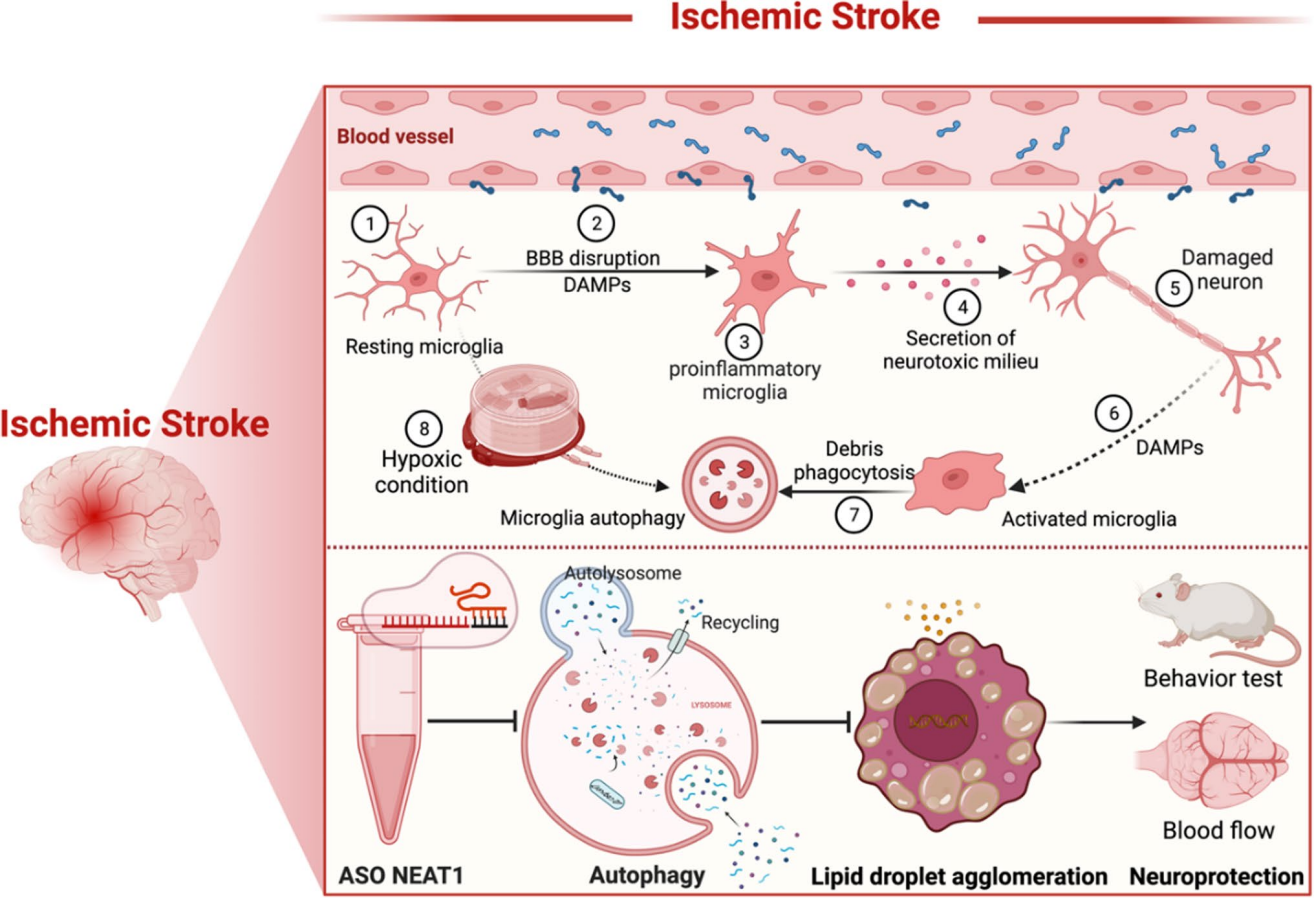

**Supplementary Information:**

The online version contains supplementary material available at 10.1007/s00018-023-05045-7.

## Introduction

Cerebral ischemia comprises a complex signaling cascade, among which microglia play a pivotal role. Upon stroke, microglia become immediately active as firstline defenders of tissue injury and migrate to the site of injury. Activated microglia stimulate various cellular signaling cascades, involving the secretion of immunomodulatory molecules such as cytokines, chemokines, and free radicals [1]. Furthermore, such microglia-associated inflammatory signaling cascades are linked to a plethora of other cellular pathways, among which autophagy. Indeed, recent data suggests that microglia would display a disturbed autophagic flux, resulting in a pro-inflammatory M1 phenotype and pronounced cell and tissue injury when exposed to in vitro hypoxia or in vivo stroke [2]. Under such conditions, activation of autophagy may reduce microglial activation and promote an M2 microglia phenotype polarization, thus inhibiting inflammasome activity and resulting in improved stroke outcome. On the contrary, excessive activation of autophagy results in cell death and exacerbates ischemic brain injury, although the precise role of (microglial) autophagy under stroke conditions remains a matter of debate [3, 4].

Recently, accumulating evidence suggests an agglomeration of lipid droplets (LD) in the brain during development and aging, but also under pathological conditions such as neurodegenerative diseases and cancer formation, particularly in microglia and astrocytes [5–9]. LD consists of a hydrophobic core of neutral lipids, mainly triacylglycerols and cholesteryl esters, enclosed by a phospholipid monolayer adorned with various proteins that govern LD activity [10]. The agglomeration of LD in microglia of the aging brain and under various neurodegenerative disease models has been recently recognized as "lipid-droplet-accumulating microglia" (LDAM). The latter display microglial dysfunction such as phagocytosis deficits, excessive production of proinflammatory cytokines, increased generation of reactive oxygen species, and decreased cholesterol export compared with neuroprotective lipopolysaccharides-activated LD-rich microglia [11]. However, few studies have focused on the formation of LD after cerebral infarction. As a matter of fact, the original article was published in 2001 illustrating magnetic resonance lipid signals in stroke rat brains correlating with neutral lipids agglomeration [12], albeit that study was per se descriptive only.

Long non-coding RNAs (lncRNA) typically exhibit a length of more than 200 nucleotides without encoding for proteins though [13]. Following ischemic stroke, abundant evidence has shown that lncRNA participate in the complex molecular process of ischemic signaling cascades and influence the ischemic injury progression by various mechanisms [14–16]. Indeed, both preclinical and clinical data describe a stroke-regulated expression of lncRNA. Among these lncRNA, the nuclear paraspeckle assembly transcript 1 (NEAT1) is a viable target for future therapy of ischemic stroke [17, 18]. NEAT1 is synthesized by RNA polymerase II from a region on the human chromosome 11q13 known as multiple endocrine neoplasias (MEN) type I [19]. Overexpression of NEAT1 exacerbates brain injury in stroke rats [20]. On the contrary, downregulation of NEAT1 protects neurons against apoptosis in preclinical stroke settings, significantly inhibiting M1 polarization of microglia [21]. Under such preclinical stroke conditions, NEAT1 can also function as a miRNA sponge to modulate the inflammatory response generated upon induction of the noxious stimulus through the miR‑374a‑5p/NFAT5 signaling pathway [22]. Interestingly, NEAT1 may also play a pivotal role for the development of atherosclerosis where blocking of NEAT1 represses lipid uptake in human macrophage THP-1 cells [23]. Despite this information available, evidence regarding the precise role of NEAT1 under stroke conditions and its interrelationship with LD formation and autophagy activation in stroke remains unclear.

The present study strived to shed novel light on the complex machinery underlying NEAT1-associated signaling pathways with regard to ischemic stroke. As such, we utilized an in vitro stroke model of microglia and an in vivo murine stroke model. An antisense oligonucleotide (ASO) was used to silence NEAT1 effectively, analyzing both LD formation and autophagy-related proteins under such conditions. To further understand the molecular mechanisms involved, 3-methyladenine (3-MA) and rapamycin (RAPA) were used to inhibit or activate autophagy, respectively, illustrating the interaction between autophagy and LD agglomeration in a NEAT1-dependent fashion.

## Materials and methods

### Cell culture

Primary microglia were isolated from neonatal C57BL/6 mice from postnatal day 0 using a traditional enzyme digestion approach with slight modification [24, 25]. Firstly, the brains were removed, and the meninges were separated. Thereafter, the cortices and hippocampi were isolated with forceps. These cortices and hippocampi were then transferred to a falcon tube and digested with trypsin. After centrifugation, the cells were collected and then suspended in DMEM medium (PAN-Biotech, Germany) supplemented with 10% fetal bovine serum (FBS, Sigma-Aldrich Chemie GmbH, Germany) and 1% penicillin/streptomycin (P/S, Fisher Scientific GmbH, Germany) before being grown in Poly-D-Lysine (PDL, Sigma-Aldrich, St. Louis, MO, USA)-coated T75 flasks in a 37 °C, 5% CO_2_ incubator. The culture medium was changed the next day to eliminate cell debris. After 5–7 days, the astrocytes established an adherent cellular layer at the bottom of the flask, with microglia growing on top of it. Purified microglia or astrocytes were collected in a conditioned culture medium (DMEM/F12 containing 10% FBS and 1% P/S for microglia, DMEM of 4.5 g/l glucose culture medium supplemented with 10% FBS, 1% GlutaMAX (100x), and 1% P/S for astrocytes) after vigorously taping the flasks on the bench top. In order to prevent contamination of a small number of astrocytes in the cultured primary microglia cells, we performed three passages before formally applying them in subsequent experiments.

Primary cortical neurons were obtained from E16.5 mouse embryos. The cortex pieces were isolated as before and trypsinized in a 37 °C waterbath for 14 min and then dissociated through fire-polished Pasteur pipettes. Neurons were cultured on PDL-coated 24-well plates at a density of 100,000/cm^2^ and grown in neurobasal medium (Gibco, Darmstadt, Germany) complemented with HEPES (Sigma-Aldrich, St. Louis, MO, USA), transferrin (Sigma-Aldrich, St. Louis, MO, USA), P/S, L-glutamine (Seromed, Dollnstein, Germany), and B27 supplement (Gibco, Darmstadt, Germany). Cells were used at 5 days in vitro.

### Establishment of oxygen-glucose-deprivation (OGD) model

The OGD model was established to create hypoxic conditions in vitro. Primary microglia were exposed to OGD when they achieved 80–90% confluency. These cells were cultivated in a glucose-free balanced salt (BSS0) solution (116 mM NaCl, 5.4 mM KCl, 0.8 mM MgSO_4_, 1 mM NaH_2_PO_4_H_2_O, 26.2 mM NaHCO_3_, 10 mM HEPES, 0.01 mM Glycine and 1.8 mM CaCl_2_, pH 7.2–7.4) and subsequently transported to a hypoxic incubator chamber with 95% N_2_, 5% CO_2_, and 70% humidity at 37 °C (Toepffer Laborsysteme GmbH, Göppingen, Germany) for different durations of time. Afterwards, the cells were removed from the hypoxic chamber and were incubated with cell culture medium with additional factors under standard cell culture conditions (reoxygenation).

### Cell transfection

ASO was used to knock down NEAT1 in primary microglia using Turbofect Transfection reagents (Thermo Fisher Scientific, Waltham, Massachusetts, USA) according to the manufacturerʹs instructions. ASO NEAT1 (5′-GGAAATCATAGAGGACAGGC-3′) or ASO scramble (5′-GAAGAAGTACGAAGTGACGC-3′) were synthesized by the INTEGRATED DNA TECHNOLOGIES company (https://www.idtdna.com). The concentration of ASO scramble used aligns with the corresponding ASO NEAT1, ranging from 20 to 80 nM.

### Cell survival

Cell viability was measured by a colorimetric assay using MTT (Thiazolyl Blue Tetrazolium Bromide, Sigma-Aldrich, St. Louis, MO, USA) as described before. The data is presented as relative changes in percent compared to untreated controls [26].

### Co-culture of microglia with primary neurons or astrocytes

A transwell system equipped with chambers (0.4 μm pore size; Greiner bio-one, Frickenhausen, Germany) was used to study the intercellular communication between microglia and primary neurons or astrocytes. This design facilitated the exchange of secreted substances between cells without requiring direct cell-to-cell contact [27]. Primary neurons or astrocytes were seeded on 24-well plates, respectively. Simultaneously, neurons underwent 10 h exposure to OGD, while astrocytes experienced 8 h of OGD [25, 28]. Concurrently, primary microglia were seeded onto a 24-well insert chamber. To illustrate whether knockdown of NEAT1 in primary microglia affects neurons and astrocytes in our oxygen-glucose-deprivation/reoxygenation (OGD/R) models, microglia were treated under four different treatment conditions (normoxia, OGD, OGD + ASO scramble, OGD + ASO NEAT1). Thereafter, microglia were then introduced into the 24-well plates containing hypoxia-treated neurons and astrocytes followed by co-cultured under normoxic conditions for 24 h.

### Middle cerebral artery occlusion (MCAO) model

All animal studies were carried out following the National Institutes of Health guidelines for the care and use of laboratory animals and approved by local government authorities. Researchers were blinded for experimental groups throughout all experiments and stages of data analysis. The mice were randomly divided into four treatment groups, and precise numbers of mice used in each group were recorded in Supplementary Table S1. MCAO was induced in male C57BL/6 mice aged 12 weeks (Charles River, Sulzfeld, Germany) to obtain a transient focal cerebral ischemia as previously described [29]. Briefly, mice were constantly anesthetized with 2% isoflurane. A 6–0 nylon silicon-coated monofilament (Doccol Corporation, Massachusetts, USA) was inserted into the right common carotid artery to occlude the right middle cerebral artery for 45 min (min). Thereafter, the filament was removed to facilitate reperfusion. During the experiment, the Laser Speckle Imaging was recorded with a flexible probe (RWD Life Science, Guangdong, China). Sham animals underwent the same procedure but without inserting the nylon filament.

### Stereotactic brain injection of ASO NEAT1 in mice

Mice were anesthetized with ketamine 10% (100 mg/kg body weight) and xylazine 2% (10 mg/kg body weight) in 0.9% NaCl (max. injection volume 10 ml/kg body weight) and then fixed in a stereotactic frame on a warming plate under deep anesthesia. After application of an eye ointment, antiseptic treatment, skin incision, and drill hole trepanation (diameter: 0.6 mm), injections were made at x (1.5 mm from the sagittal suture), y (− 2.0 mm from the bregma), and z (2.5 mm depth) via a motorized injector (Stoelting Wood Dale, IL, USA). ASO NEAT1 (2 nmol, 2 μl) or ASO scramble (2 nmol, 2 μl) was slowly injected over 5 min using a 10 μl Hamilton cannula (outer diameter 240 μm; injection rate: 400 nl/min) once a week for 2 consecutive weeks before MCAO surgery (thus two injections in total). After the injection, the cannula remained in the tissue for 4 min to prevent reflux through the injection channel. The stereotactic injection paradigms essentially followed a protocol described by Jin et al. [17].

### Quantitative real-time PCR analysis

Total RNA was isolated using TRIzol (Invitrogen, Waltham, Massachusetts, USA) as directed by the manufacturer's instructions. The KAPA SYBR^®^ FAST One-Step Kit for LightCycler^®^ 480 (Merck Group, Darmstadt, Germany) was used to perform qRT-PCR according to the manufacturer's instructions, and the PCR primers were purchased from Eurofins Genomics (Ebersberg, Germany). The relative quantity levels were calculated with the 2^−ΔΔCt^ method using PPIA as the internal standard control. Details of the sequence can be found in Supplementary Table S2.

### Immunohistochemistry and immunocytochemistry staining

Mice were euthanized and perfused transcardially with cold phosphate-buffered saline (PBS). Brain was explanted and fixed overnight in 4% paraformaldehyde and subsequently dehydrated with 30% sucrose at 4 °C [30]. Then, the ischemic brains were paraffin-embedded, cut into 16 µm cryostat sections, and blocked with tris-buffered saline (TBS) containing 10% DS (donkey serum), 2% BSA (bovine serum albumin), and 0.25% Triton X-100 at room temperature (RT) for 1 h. For BODIPY staining, the sections were stained with BODIPY 493/503 (D3922, 1 mg/ml, Thermo Fisher Scientific, Waltham, Massachusetts, USA) for 30 min. BODIPY is diluted using TBS. Thereafter, the sections were washed 3 times with TBS followed by 4ʹ,6-Diamidin-2-phenylindol (DAPI, 1:10,000; AppliChem, Darmstadt, Germany) staining.

For cells, the slides were fixed with 4% paraformaldehyde and permeabilized with 0.25% PBS-Triton X-100 and then 10% DS with 1% BSA was used for blocking. The primary antibody (polyclonal rabbit anti-CD11b antibody, 2 µg/ml, Abcam, UK; monoclonal rat anti-CD68 antibody, 2 µg/ml, BioRad, USA; polyclonal rabbit CX3CR1 antibody, 2 µg/ml, Thermo Fisher Scientific, USA; polyclonal rabbit anti-Iba1 antibody, 2 µg/ml, Abcam, UK; polyclonal rabbit anti-NeuN antibody, 2 µg/ml, Abcam, UK; polyclonal chicken anti-GFAP antibody, 2 µg/ml, Millipore, USA) was incubated at 4 °C overnight. The Cy-3 labeled or Alexa Fluor 488 labeled secondary antibody (1:10,000, Jackson Immuno, West Grove, PA, USA) was used to detect the primary antibody staining. ImageJ (National Institutes of Health, Bethesda, USA was used for cell counting and intensity quantification. The negative and unstained controls of staining for animals and cells are shown in the Supplementary file.

### Western blot

The protein samples were harvested from brain tissues or cultured cells with radioimmunoprecipitation assay (RIPA) solution, and protein concentrations were quantified using the Pierce BCA Protein Assay Kit (Thermo Fisher Scientific, Waltham, Massachusetts, USA). Thereafter, proteins were separated on 10% or 15% SDS-PAGE and transferred to polyvinylidene fluoride membranes (Biorad, Hercules, California, USA). Subsequently, the membranes were blocked with 5% skim milk dissolved in deionized water and incubated with the primary antibodies overnight at 4 °C. On the second day, the blots were incubated with horseradish peroxidase (HRP) coupled secondary antibody for 1 h at RT. The specific antibody working dilutions are given in Supplementary Table S3. After that, the rinsed membranes were immersed in ECL chemiluminescence reagent before the blots were exposed to the imaging equipment ChemiDoc station (Biorad). Image Lab Software was used to evaluate the data further.

### Analysis of post-stroke motor coordination deficits

To guarantee proper test behavior, mice were trained on days 1 and 2 before the induction of stroke. Testing was performed on post-stroke day 7 using the rotarod test, tightrope test, balance beam test, and the paw slips recording, as previously described [30]. The details can be found in Supplementary Materials and Methods S1 and Table S4.

### Laser speckle imaging for cerebral perfusion analysis

At the 7th day after the occurrence of ischemia, the cortical blood flow was examined using continuous Laser Speckle Imaging through the exposed skull while the mice were under anesthesia. The blood flow in the ipsilateral and contralateral sides of the cortex was assessed. Consistent positioning of the region of interest was maintained throughout the analysis, and the average measured values were collected at the same time point.

### Statistical analysis

GraphPad Prism 9 software (GraphPad Software Inc., San Diego, CA, USA) was adopted for statistical analysis and graphic production, whereas Excel was utilized for data collection. The Student's *t*-test was used to compare between two groups. A one-way ANOVA followed by Tukey's post-hoc-test was explicitly chosen to compare multiple groups. The effect size was between 0.3 and 0.4, depending on the analysis chosen. All measurement results were summarized as mean ± standard deviation, and *p* values less than 0.05 were considered significant.

## Results

### OGD induces upregulation of NEAT1 in primary microglia

**Fig. 1:**
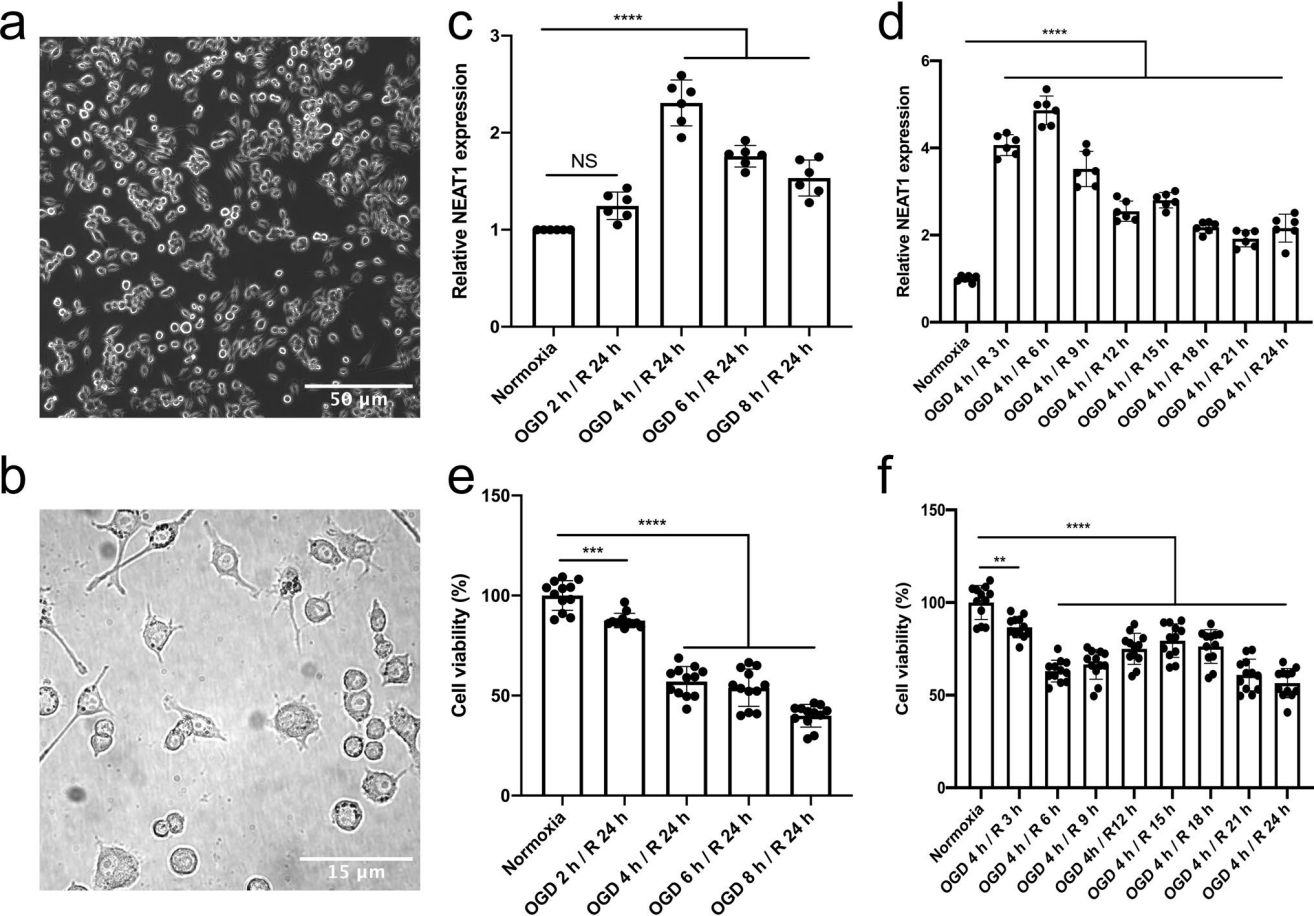
Light microscopy of primary microglia and exposure towards OGD with subsequent NEAT1 expression and cell viability analyses. Primary microglia were isolated from cerebral cortices and hippocampi of newborn WT C57BL/6 J mice. **a** Light microscopy of primary microglia in culture. **b** The magnified image of primary microglia under bright-field microscopy. **c**, **d** The corresponding NEAT1 expression at different time points of OGD and reoxygenation in comparison to PPIA. **e**, **f** The MTT assay was used to evaluate the cell viability after various OGD and reoxygenation time points. All results are expressed as mean ± standard deviation and analyzed by one-way ANOVA followed by Tukey's post-hoc-test. NS, no significance, **p < 0.01, ***p < 0.001, ****p < 0.0001. Abbreviations: NEAT1, nuclear paraspeckle assembly transcript 1; OGD, oxygen-glucose-deprivation; PPIA, peptidylprolyl isomerase A

Before starting OGD experiments, cell morphology and expression patterns of four well-defined markers were analyzed in order to confirm a microglial phenotype (Figs. 1a, b, S1). As such, primary microglia showed a ramified morphology in the bright-field microscope, expressing CD11b, CX3CR1, Iba1, and CD68. To observe the role of NEAT1 in these cells under hypoxic conditions, we first explored the expression of NEAT1 in microglia under different time points of OGD (2, 4, 6, or 8 h) followed by 24 h of reoxygenation. As a matter of fact, hypoxia significantly increased NEAT1 expression peaking after 4 h of OGD when compared to normoxic controls (Fig. 1c). Setting the OGD duration at 4 h, the following experiments analyzed NEAT1 levels after 4 h of OGD with subsequent reoxygenation periods of 3, 6, 9, 12, 15, 18, 21, or 24 h, respectively. Under these conditions, NEAT1 expression in primary microglia peaked at 6 h of reoxygenation (Fig. 1d). For the remainder of the study, the experimental in vitro paradigm was set at 4 h of OGD with 6 h of reoxygenation. Primary microglia were not only used for the analysis of NEAT1 expression, however, but also for the measurement of cell viability under the aforementioned OGD conditions. Hence, cell viability rates corresponded with the duration of OGD and reoxygenation as given in Fig. 1e, f.

### Knockdown of NEAT1 affects expression patterns of LD in primary microglia exposed to hypoxia

**Fig. 2:**
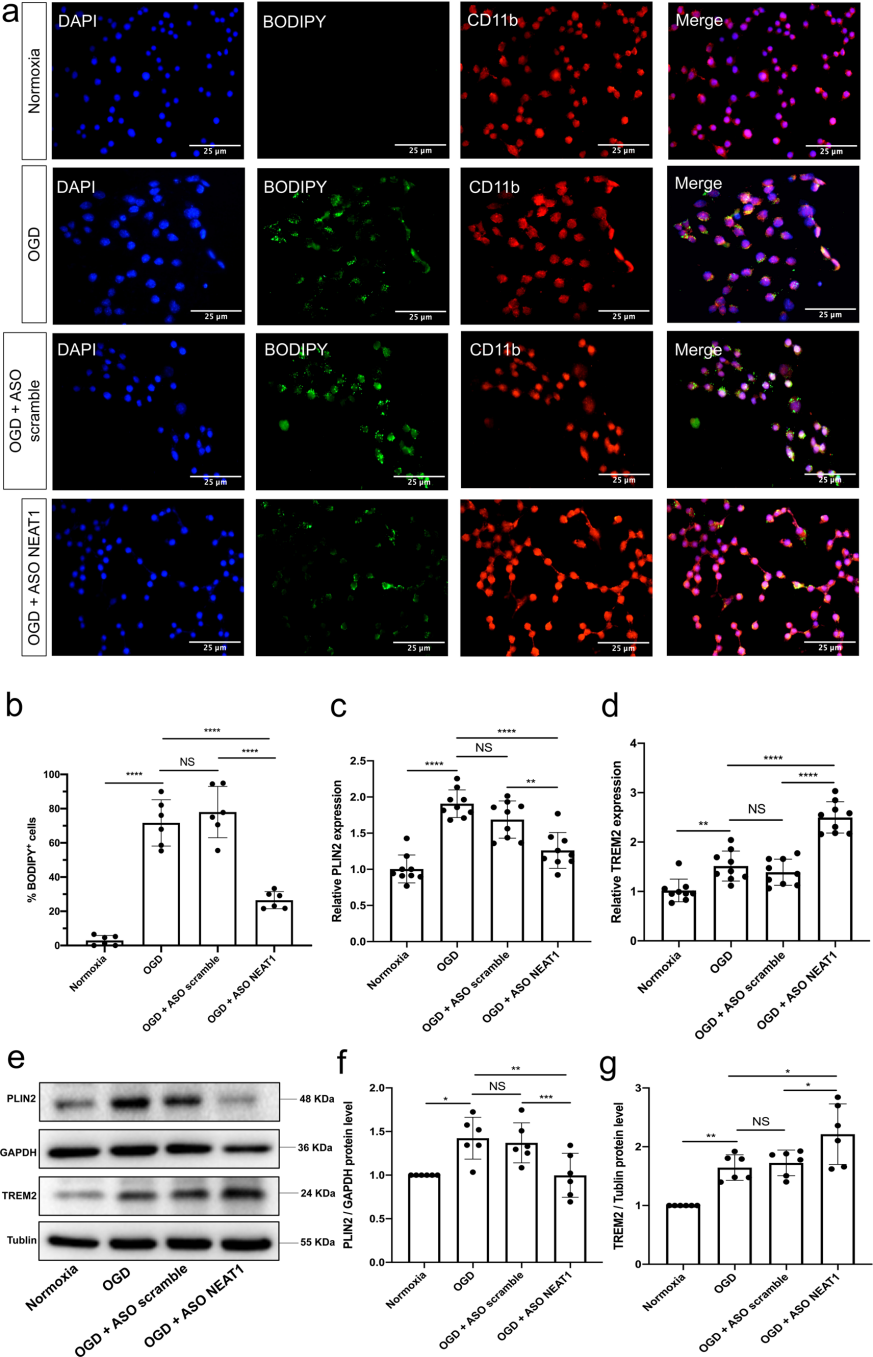
Knockdown of NEAT1 affects expression patterns of LD in primary microglia exposed to hypoxia. **a** The fluorescence staining of neutral LD (BODIPY, green) in microglial cells (CD11b, red) cultured with ASO scramble or ASO NEAT1. **b** Statistical analysis of the percentage of LD in whole cells. **c**, **d** Primary microglia were transfected with ASO NEAT1 (or ASO scramble as control) for 24 h to generate a stable NEAT1 knockdown. The expression of the LD-related genes PLIN2 (**c**) and TREM2 (**d**) was detected using qRT-PCR in comparison to PPIA. **e**–**g** Western blot analysis of PLIN2 and TREM2 expression in OGD/R microglial cells after treatment with ASO scramble or ASO NEAT1. The relative quantitative analysis was normalized against GAPDH or tublin, as indicated. All results are expressed as mean ± standard deviation and analyzed by one-way ANOVA followed by Tukey's post-hoc-test. NS, no significance, *p < 0.05, **p < 0.01, ***p < 0.001, and ****p < 0.0001. Abbreviations: NEAT1, nuclear paraspeckle assembly transcript 1; ASO, antisense oligonucleotide; OGD/R, oxygen-glucose-deprivation/reoxygenation; LD, lipid droplets; PLIN2, perilipin 2; TREM2, triggering receptor expressed on myeloid cells 2; PPIA, peptidylprolyl isomerase A

To further explore the role of NEAT1 in microglia exposed to hypoxia, antisense oligonucleotides downregulating NEAT1 (ASO NEAT1) or negative controls (ASO scramble) were used to transfect primary microglia (Fig. S2a). Establishing optimal knockdown conditions, different concentrations of ASO NEAT1 were used and the inhibition efficiency was assessed by qRT-PCR, thereafter. Expression of NEAT1 was most effectively inhibited under physiological conditions when microglia were treated with 80 nM of ASO NEAT1 (Fig. S2b). Effective knockdown of NEAT1 in these cells significantly increased the viability of microglia under OGD conditions when compared to the controls (Fig. S2c). Subsequently, BODIPY staining was used in order to elucidate whether or not NEAT1 expression patterns affect LD agglomeration in microglia under hypoxic conditions (Fig. 2a, b). The latter yielded an upregulation of LD formation in microglia when compared to normoxic conditions. In contrast, the concurrent transfection with ASO NEAT1 significantly inhibited LD expression under these conditions. Additional qRT-PCR experiments revealed an upregulation of the LD-related proteins perilipin 2 (PLIN2) and triggering receptor expressed on myeloid cells 2 (TREM2) upon OGD induction. Interestingly, NEAT1 silencing resulted in a differential regulation of both PLIN2 and TREM2 expression under OGD conditions. Whereas PLIN2 expression was significantly reduced, TREM2 expression was increased even further due to NEAT1 knockdown (Fig. 2c, d). Western blot experiments confirmed the aforementioned qRT-PCR results using the very same conditions (Fig. 2e–g). These results suggest that lncRNA NEAT1 affects expression patterns of LD and regulates the expression of PLIN2 as well as TREM2 in primary microglia exposed to hypoxic cell injury.

### Knockdown of NEAT1 affects signaling cascades related to autophagy in primary microglia exposed to OGD

**Fig. 3:**
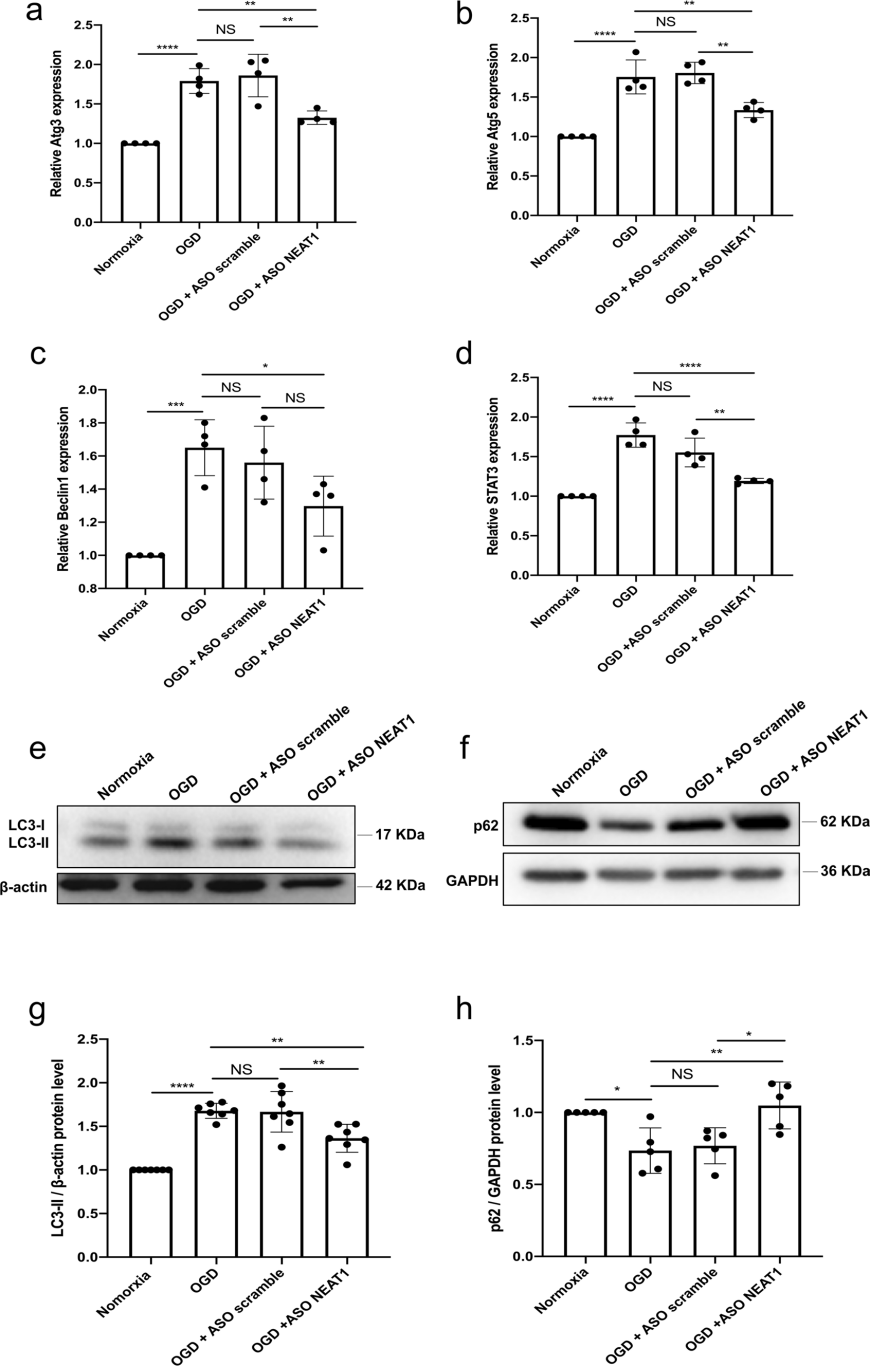
Knockdown of NEAT1 affects signaling cascades related to autophagy in primary microglia exposed to hypoxia. **a**–**d** Autophagy-related genes, i.e., Atg3 (**a**), Atg5 (**b**), Beclin1 (**c**), and STAT3 (**d**) were determined by quantitative qRT-PCR normalized to PPIA. **e**–**h** NEAT1 knockdown repressed LC3 but activated p62 expressions. Western blot was utilized to analyze the expression of LC3 (**e**) at a 17 kDa band and p62 (**f**) at a 62 kDa band in microglial cells in four parallel groups (normoxia, OGD, OGD + ASO scramble, and OGD + ASO NEAT1). The relative quantitative analysis was normalized against β-actin or GAPDH. Results are expressed as mean ± standard deviation and analyzed by one-way ANOVA followed by Tukey's post-hoc-test. NS, no significance, *p < 0.05, **p < 0.01, ***p < 0.001, and ****p < 0.0001. Abbreviations: NEAT1, nuclear paraspeckle assembly transcript 1; ASO, antisense oligonucleotide; OGD, oxygen-glucose-deprivation; Atg3, autophagy-related 3; PPIA, peptidylprolyl isomerase A

In light of autophagy being an important signaling cascade in hypoxic and ischemic settings, we next explored the impact of NEAT1 on this particular pathway. Primary microglia were again transfected with either ASO scramble or ASO NEAT1 and then subjected to OGD conditions, as stated before. Induction of OGD yielded increased mRNA levels of autophagy-related 3 (Atg3), Atg5, Beclin1, and STAT3 compared to the normoxia group, indicating an activation of autophagy upon hypoxia induction in primary microglia. When cells were transfected with the ASO NEAT1, however, the expression of these autophagy-related proteins Atg3, Atg5, Beclin1, and STAT3 was markedly reduced on the mRNA level (Fig. 3a–d). In line with this, further analysis of the autophagy-associated proteins LC3 and p62 revealed a decreased and increased expression respectively of these proteins in hypoxic microglia where NEAT1 was knocked down (Fig. 3e–h). These findings demonstrate that knockdown of NEAT1 impairs hypoxia-induced activation of autophagy in primary microglia.

### Regulation of the autophagy affects LD formation in primary microglia under hypoxic conditions

**Fig. 4:**
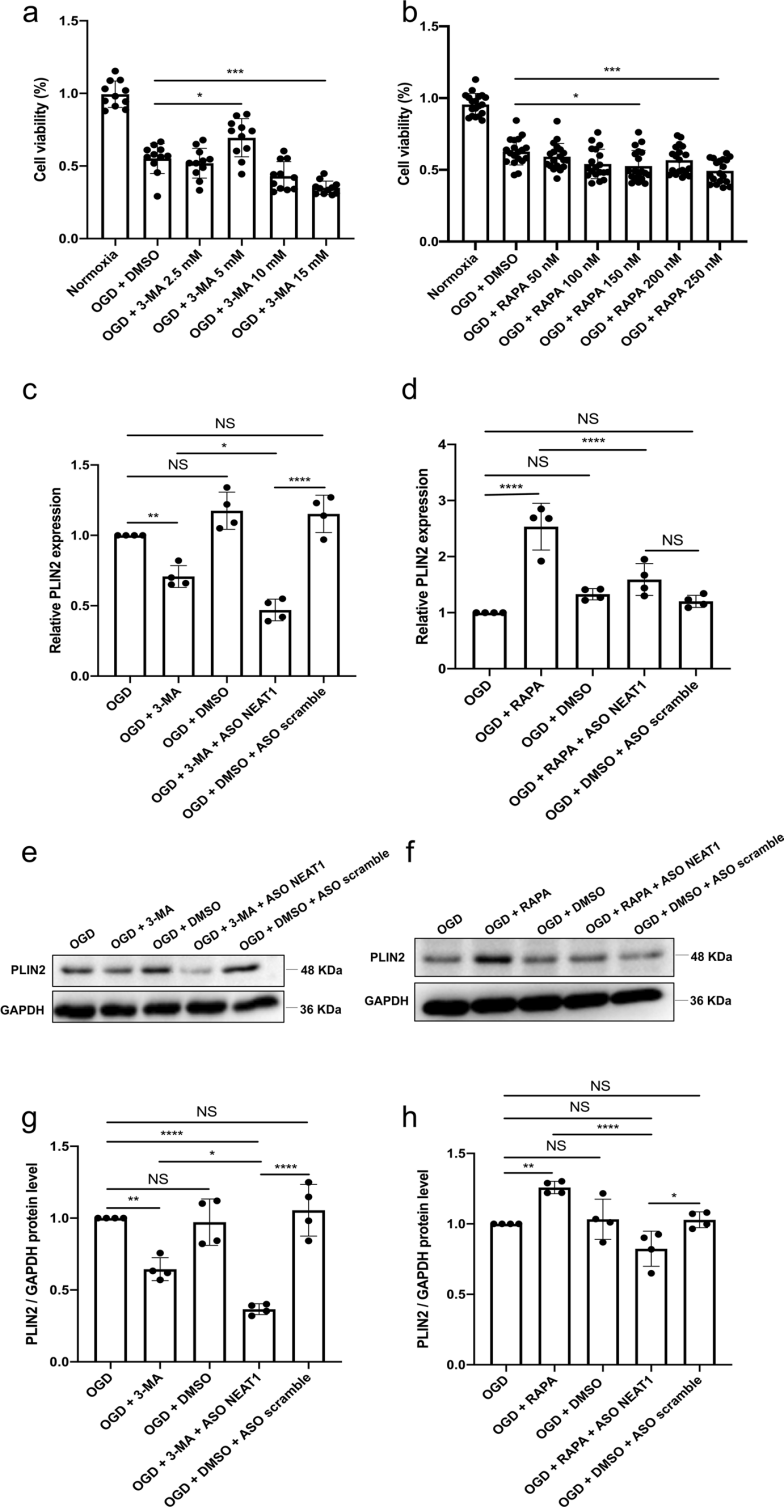
Regulation of autophagy affects PLIN2 expression in primary microglia exposed to OGD. **a**, **b** The impact of autophagy on microglia survival after OGD was evaluated using different concentrations of the autophagy inhibitor 3‐MA or autophagy stimulator RAPA in comparison to microglia treated with the solvent DMSO using the MTT assay. **c**, **d** Relative PLIN2 expressions in microglial cells are shown after inhibition (**c**) or activation (**d**) of autophagy under OGD conditions using qRT-PCR normalized to PPIA. **e**–**h** Protein levels of PLIN2 in microglia are displayed after inhibition or activation of the autophagy pathway under OGD conditions. PLIN2 protein was recognized as a 48 kDa band, and GAPDH (36 kDa) was used as a reference. All results are expressed as mean ± standard deviation and analyzed by one-way ANOVA followed by Tukey's post-hoc-test. NS, no significance, *p < 0.05, **p < 0.01, ***p < 0.001, and ****p < 0.0001. Abbreviations: ASO, antisense oligonucleotide; OGD, oxygen-glucose-deprivation; 3-MA, 3-methyladenine; RAPA, rapamycin; PLIN2, perilipin 2; DMSO, dimethyl sulfoxide; PPIA, peptidylprolyl isomerase A

To further illustrate the interaction between autophagy and LD agglomeration, different concentrations of 3-MA (inhibitor) and RAPA (stimulator) were used to regulate autophagy in primary microglia exposed to OGD. Microglia viability was initially increased due to the application of 3-MA in a dose-dependent manner, although 3-MA showed toxic side effects at concentrations beyond 5 mM (Fig. 4a). On the contrary, incubation with RAPA at 150 nM diminished cell survival rates significantly (Fig. 4b). Therefore, concentrations of 5 mM for 3-MA and 150 nM for RAPA were used as optimal concentrations for interfering with autophagy in primary microglia under OGD conditions. When analyzing expression patterns of the LD-related protein PLIN2 in the different OGD groups, application of 3-MA significantly reduced mRNA levels of PLIN2 in microglia. Expression of PLIN2 was decreased even further when microglia were transfected with ASO NEAT1 (Fig. 4c). The autophagy stimulator RAPA, in turn, significantly increased PLIN2 expression in microglia exposed to OGD, a process that was reversed due to the silencing of NEAT1 (Fig. 4d). The suppressive action of NEAT1 on OGD/R-induced LD agglomeration was further confirmed by western blot assay. As shown in Fig. 4e–h, decreased expression of PLIN2 was found in hypoxic cells where NEAT1 was downregulated. These results suggest that downregulation of NEAT1 promotes the inhibitory effect of 3-MA on LD agglomeration after the inhibition of autophagy.

**Fig. 5:**
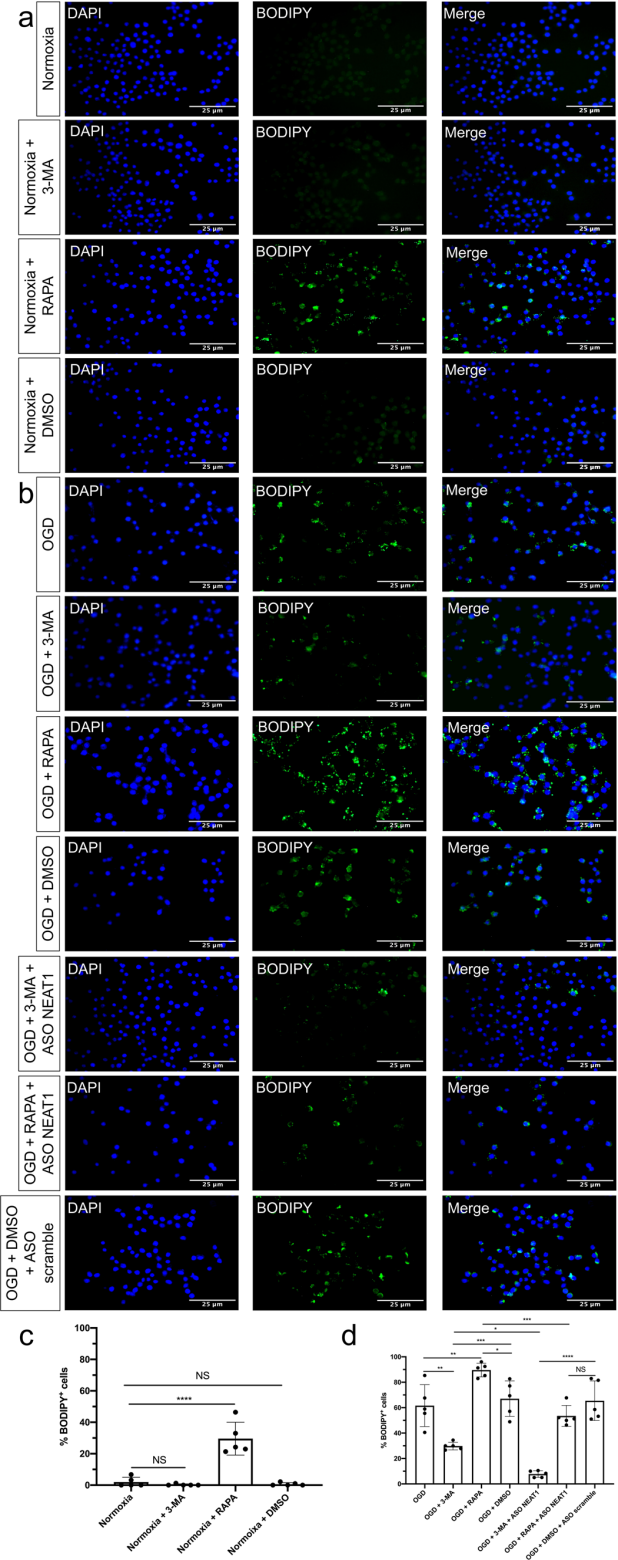
Regulation of autophagy affects LD formation in primary microglia. **a** Microglial cells were treated with 3-MA or RAPA under normoxia conditions, followed by BODIPY staining. **b** Microglia were treated with 3-MA or RAPA with additional ASO scramble or ASO NEAT1 treatment under hypoxia conditions, followed by BODIPY staining. **c**, **d** Quantification analysis of the percentage of LD in whole cells. All results are expressed as mean ± standard deviation and analyzed by one-way ANOVA followed by Tukey's post-hoc-test. NS, no significance, *p < 0.05, **p < 0.01, ***p < 0.001, and ****p < 0.0001. Abbreviations: LD, lipid droplets; 3-MA, 3-methyladenine; RAPA, rapamycin; ASO, antisense oligonucleotide; NEAT1, nuclear paraspeckle assembly transcript 1

The results shown before indicated a NEAT1-dependent regulation of LD formation in primary microglia exposed to OGD. Hence, BODIPY staining was performed to label LD in microglia under both normoxic and hypoxic conditions. Under normoxic conditions, stimulation of autophagy by using RAPA gave rise to an increased BODIPY fluorescence intensity in comparison to controls; inhibition of autophagy did not affect fluorescent intensity under these conditions (Fig. 5a, c). Nevertheless, the number of BODIPY^+^ cells was significantly decreased when microglia were treated with 3-MA under OGD conditions, wheres RAPA initiated an increased level of BODIPY fluorescence intensity. Knockdown of NEAT1 also gave rise to decreased LD formation in hypoxic microglia as indicated by BODIPY staining, a process that was enhanced even further when such cells were pretreated with 3-MA (Fig. 5b, d).

**Fig. 6:**
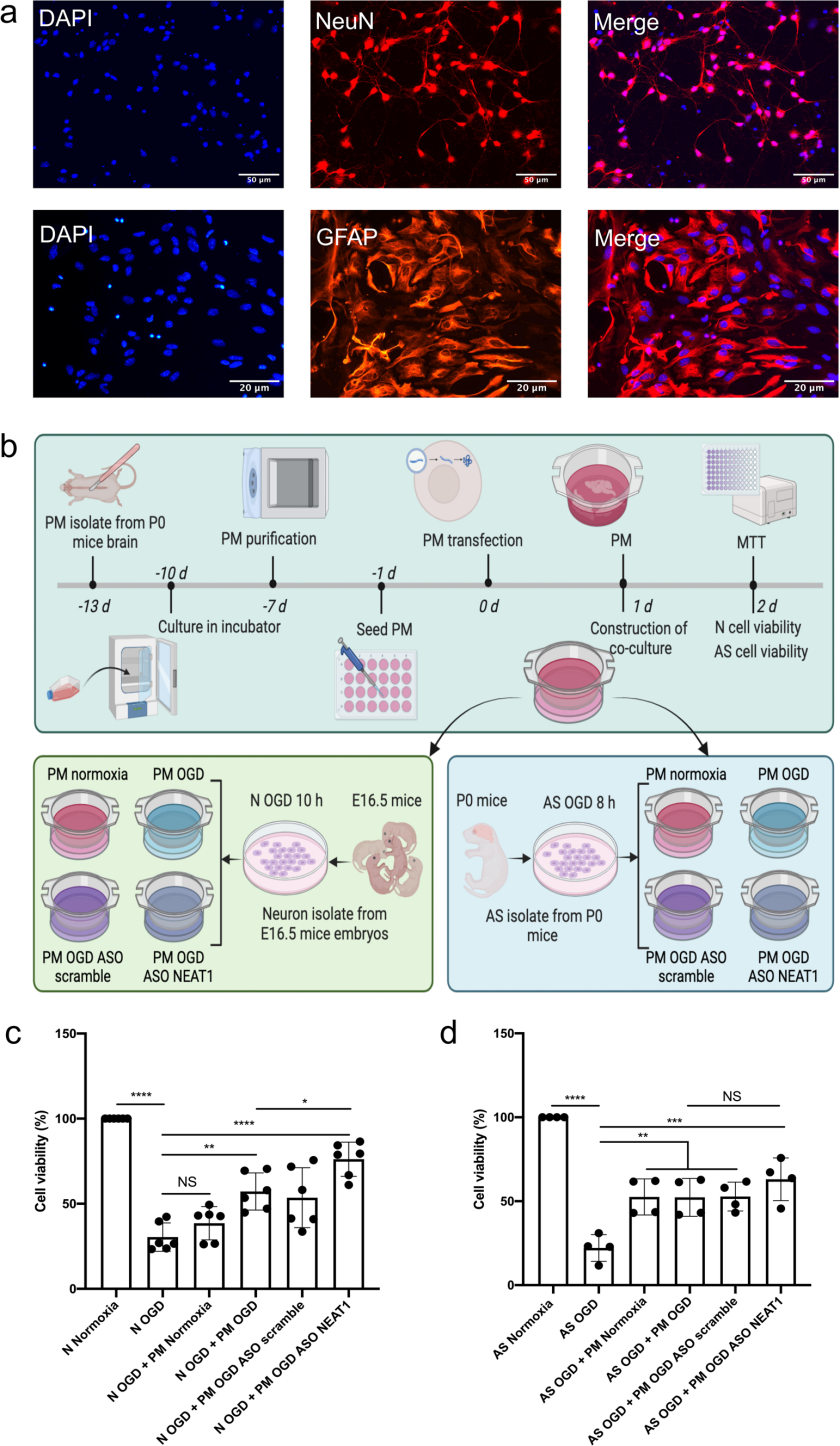
Knockdown of NEAT1 in microglia protects primary neurons from OGD injury. **a** Immunofluorescence images of primary neurons (NeuN) and astrocytes (GFAP) in red under normoxia conditions. Nuclei were counterstained with DAPI in blue. **b** A summary of the experimental paradigm for the in vitro co-culture model. Primary microglia and astrocytes were isolated from neonatal C57BL/6 mice on postnatal day 0, whereas primary cortical neurons were obtained from E16.5 mouse embryos. The neurons were subjected to 10 h of OGD, whereas astrocytes underwent 8 h of OGD. Microglia were subjected to four conditions (normoxia, OGD, OGD + ASO scramble, OGD + ASO NEAT1), followed by co-culture with hypoxic neuorns or astrocytes. Afterwards, they were cultured under normoxic conditions for 24 h. **c**, **d** The MTT assay was performed to assess the cell survival of primary neurons and astrocytes co-cultured with microglia, which were treated in different ways. As such, microglia were treated with ASO NEAT1 knockdown constructs or with scramble constructs. All results are expressed as mean ± standard deviation and analyzed by one-way ANOVA followed by Tukey's post-hoc-test. NS, no significance, *p < 0.05, **p < 0.01, ***p < 0.001, and ****p < 0.0001. Abbreviations: NEAT1, nuclear paraspeckle assembly transcript 1; ASO, antisense oligonucleotide; OGD, oxygen-glucose-deprivation; PM, primary microglia; N, neuron; AS, astrocyte

To further elucidate whether knockdown of NEAT1 in primary microglia affects neurons and astrocytes in our OGD/R models, we established a microglia-neuron or microglia-astrocyte co-cultured model. In this co-culture system, we co-cultured primary neurons or astrocytes with microglia that were transfected with ASO scramble or ASO NEAT1 (Fig. 6a, b). In accordance with MTT results, a significant promotion of viability was observed in neurons when co-cultured with microglia in which NEAT1 was knocked down (Fig. 6c). Unlike the neurons, however, NEAT1 knockdown in microglia did not have an impact on astrocyte viability (Fig. 6d). These findings highlight the potential importance of NEAT1 in modulating neuronal survival and shed light on the complex interplay between NEAT1, neurons, and neuroglial cells in the context of hypoxia.

### Silencing of NEAT1 ameliorates LD agglomeration and reduces activation of autophagy under in vivo stroke conditions

**Fig. 7:**
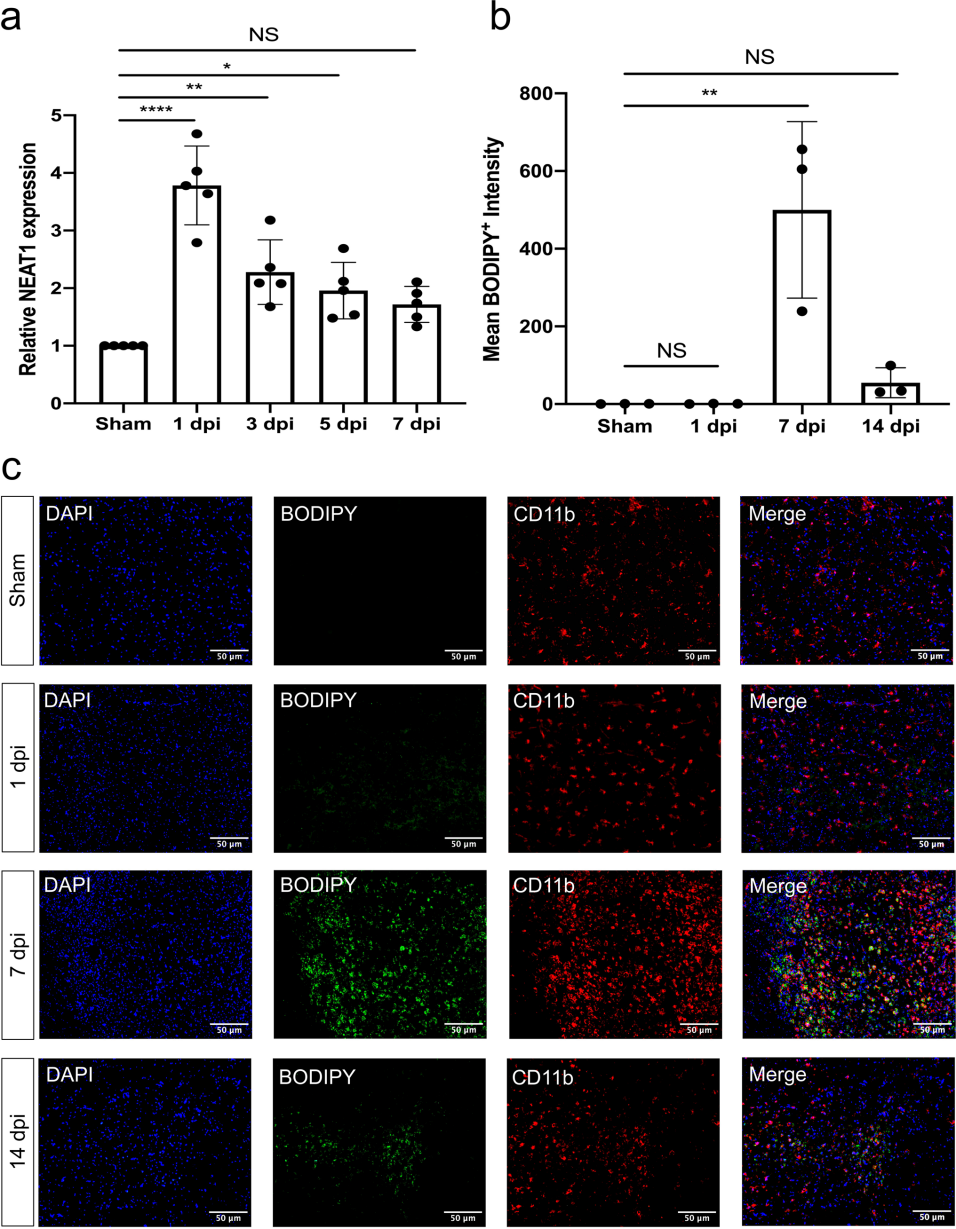
NEAT1 and LD expression in stroke mice. **a** The expression of NEAT1 on different days following cerebral ischemia in comparison to PPIA. **b** Quantification of the fluorescence intensity expression of BODIPY. **c** Images show the dual staining for BODIPY and CD11b in the ischemic hemisphere taken from sham and different days post-ischemic reperfusion. Results are expressed as mean ± standard deviation and analyzed by one-way ANOVA followed by Tukey's post-hoc-test. NS, no significance, *p < 0.05, **p < 0.01, and ****p < 0.0001. Abbreviations: NEAT1, nuclear paraspeckle assembly transcript 1; dpi, day post-ischemia; PPIA, peptidylprolyl isomerase A

Knockdown of NEAT1 affects both LD formation and autophagy in primary microglia exposed to hypoxia, with autophagy regulating LD formation on its own. However, inhibition or activation of the autophagy pathway did not affect the expression of NEAT1, as shown in Fig. S3. To assess the role of NEAT1 in ischemic stroke under in vivo conditions, an MCAO model was used in male mice. Upon induction of stroke, mice displayed a temporal profile of NEAT1 expression within the ischemic tissue, with a peak at 1 day post-ischemia (dpi) (Fig. 7a). LD agglomeration was also increased upon stroke induction in a time-dependent manner, albeit with a temporal shift towards later time points, i.e., LD agglomeration peaked at 7 dpi (Fig. 7b, c). Hence, further studies were done using 7 dpi as the peak of LD agglomeration in order to study the effect of NEAT1 under stroke conditions.

**Fig. 8:**
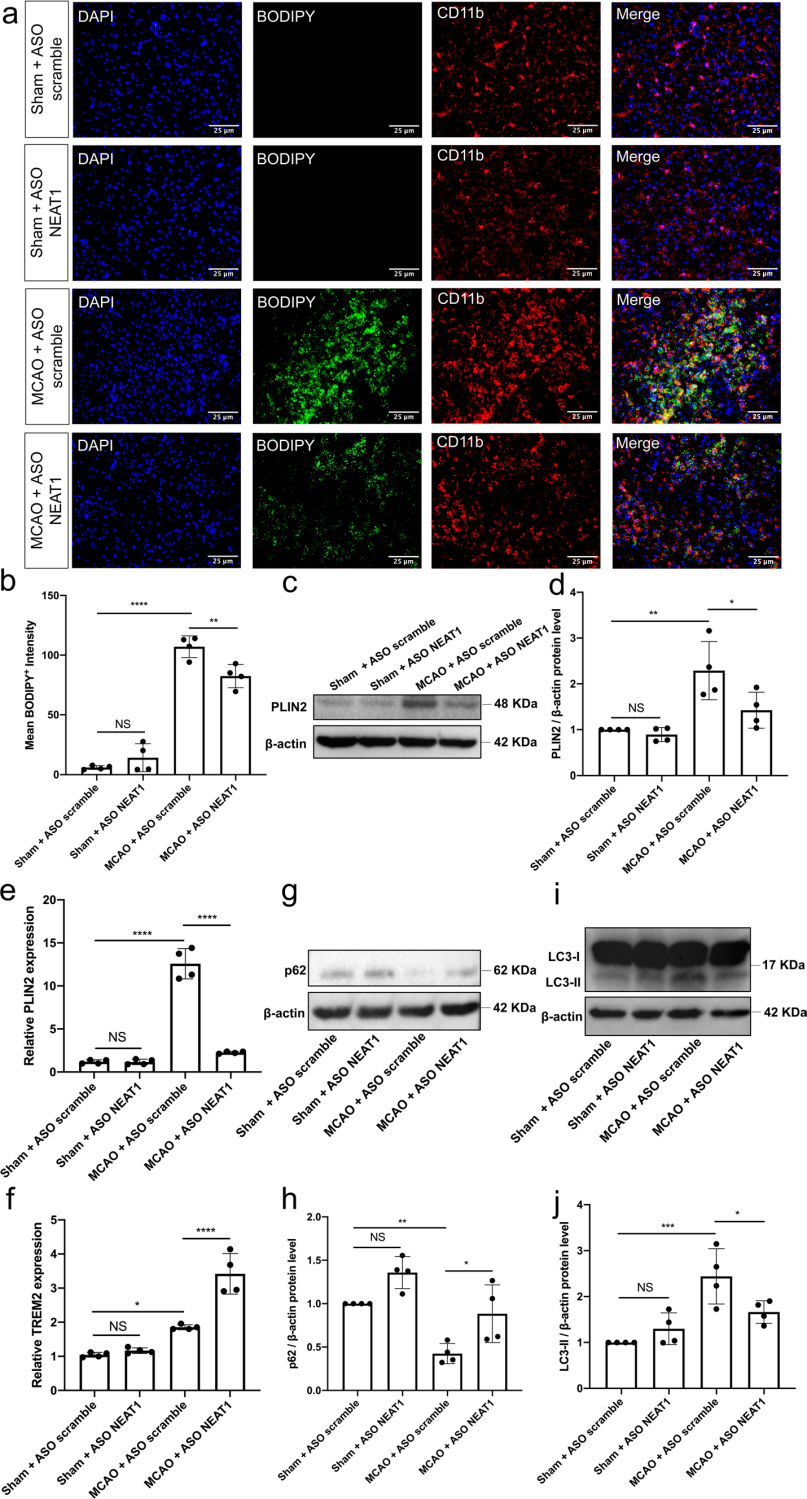
NEAT1 knockdown represses LD agglomeration and inhibits autophagy following MCAO in vivo. Mice were exposed to 45 min of MCAO followed by 7 days of survival. Sham group mice underwent the surgery procedure without MCAO. **a** Images show the dual fluorescence staining for BODIPY and CD11b in the ischemic hemisphere taken from the sham group, the negative control group (MCAO + ASO scramble) or mice treated with ASO NEAT1 at 7 dpi. Quantification of the mean BODIPY^+^ fluorescence intensity expressions between each group of mice is shown in **b**. **c**, **d** Western blot analysis of PLIN2 in whole ipsilateral cortex tissues and β-actin was used as a reference. **e**–**f** The expression of PLIN2 and TREM2 in the ipsilateral ischemic cerebral cortex from sham-operated mice (sham + ASO scramble, sham + ASO NEAT1) or from mice at 7 dpi (MCAO + ASO scramble, MCAO + ASO NEAT1) was examined by qRT-PCR. **g**–**j** Representative images of western blots are shown for the expression of p62 (**g**) and LC3 (**i**) in whole ipsilateral cortex tissues. Bar graphs show the quantitative analyses of western blots as ratios of p62/β-actin (**h**) and LC3/β-actin (**j**). All results are expressed as mean ± standard deviation and analyzed by one-way ANOVA (n = 4 mice per experimental group) followed by Tukey's post-hoc-test. NS, no significance, *p < 0.05, **p < 0.01, ***p < 0.001, and ****p < 0.0001. Abbreviations: LD, lipid droplets; ASO, antisense oligonucleotide; MCAO, middle cerebral artery occlusion; PLIN2, perilipin 2; TREM2, triggering receptor expressed on myeloid cells 2; NEAT1, nuclear paraspeckle assembly transcript 1; dpi, day post-ischemia

To further ascertain the regulatory role of NEAT1 in the pathophysiology of ischemic stroke, stereotactic injections of ASO NEAT1 were performed in order to reduce the expression of NEAT1 in the process. Two weeks after injection of either ASO scramble or ASO NEAT1, mice were subjected to MCAO and sacrificed on dpi 7. As described for the in vitro experimental series before, induction of stroke yielded increased BODIPY-labeled LD agglomeration when compared to sham mice. Knockdown of NEAT1 had no effect on LD formation in the sham groups, whereas NEAT1 knockdown gave rise to reduced LD formation in stroke mice (Fig. 8a, b). Both qRT-PCR and western blotting confirmed the aforementioned data of the BODIPY staining, i.e., PLIN2 expression was significantly decreased after NEAT1 knockdown in stroke mice (Fig. 8c–e). In addition, NEAT1 downregulation significantly upregulated the expression of TREM2 in stroke animals (Fig. 8f). Furthermore, the post-stroke effect of NEAT1 on autophagy was also studied. Consistently, the expression of the autophagosome protein LC3 was decreased, whereas p62 expression was enhanced in NEAT1 knockdown mice after MCAO (Fig. 8g–j).

### Silencing of NEAT1 alleviates ischemic brain injury in MCAO mice

**Fig. 9:**
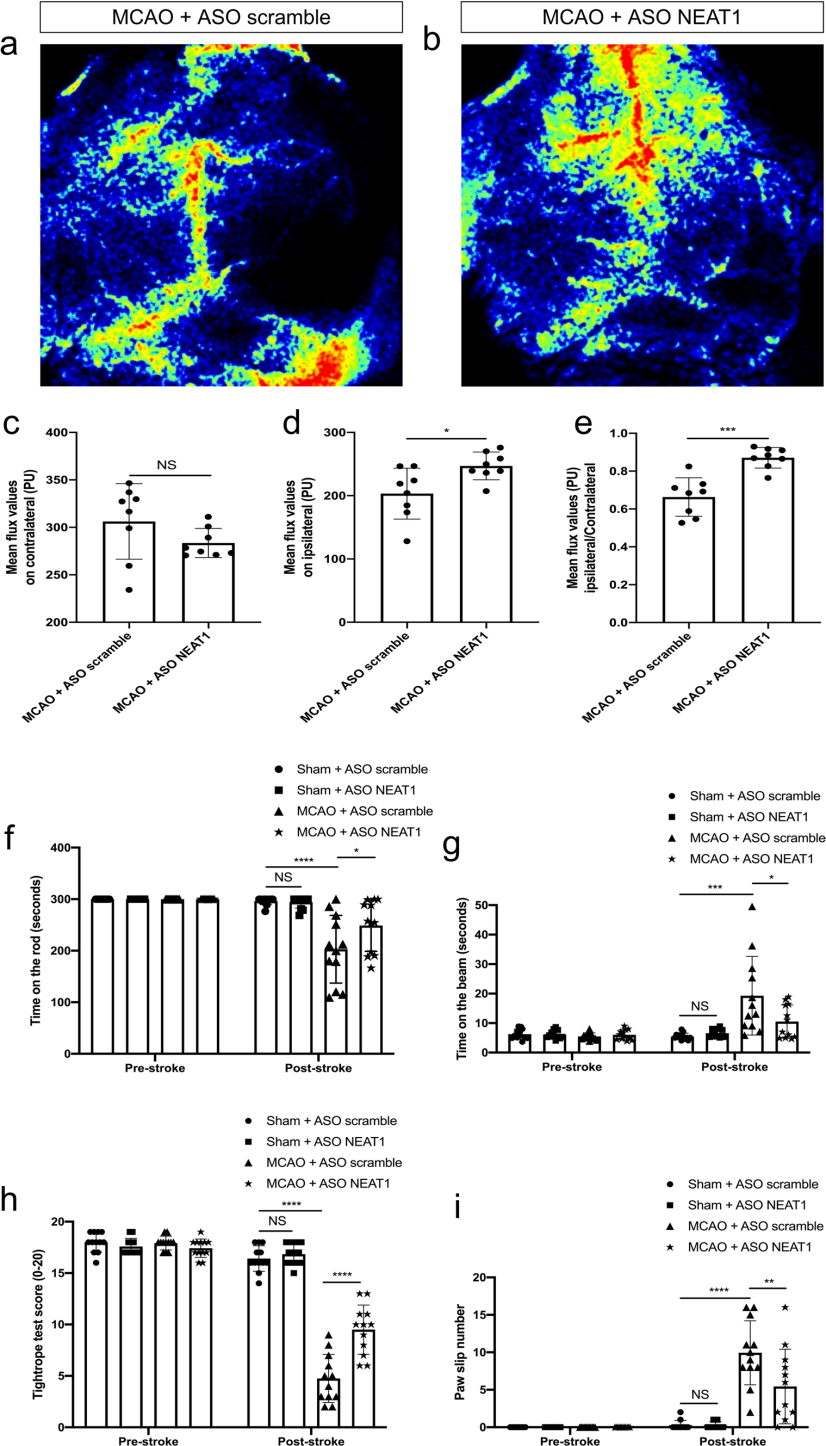
Silencing NEAT1 protects against ischemia-induced brain damage. All mice were exposed to 45 min of MCAO. **a**, **b** Laser speckle images show the effect of ASO scramble or ASO NEAT1 on cortical blood circulation at 7dpi. **c**–**e** Statistical analysis of laser speckle measurements is shown for the contralateral cortex, the ipsilateral cortex, and for the ratio ipsilateral to contralateral in mice exposed to MCAO at 7dpi.** f**–**i** Knockdown of NEAT1 reduces post-ischemic motor coordination impairment, which was evaluated using the test of rotarod (**f**), the balance beam (**g**), the tightrope (**h**), and the paw slip (**i**) on day 1 before the stroke and day 7 after the stroke. All results are expressed as mean ± standard deviation and analyzed by Student's *t*-test or two-way ANOVA (n = 8 mice per experimental group for laser speckle measurements and 12 mice for behavior tests) followed by Tukey's post-hoc-test. NS, no significance, *p < 0.05, **p < 0.01, ***p < 0.001, and ****p < 0.0001. Abbreviations: ASO, antisense oligonucleotide; MCAO, middle cerebral artery occlusion; NEAT1, nuclear paraspeckle assembly transcript 1; dpi, day post-ischemia

To investigate the effect of ASO NEAT1 on cerebral infarction, we measured cortical cerebral blood flow and neurological deficits after MCAO injury. As illustrated in Fig. 9a–c, laser speckle images showed no difference in the mean flux values of contralateral hemispheres between MCAO + ASO NEAT1 (knockdown stroke animals) and MCAO + ASO scramble groups (control stroke animals). Interestingly, knockdown of NEAT1 significantly increased cerebral perfusion in stroke animals (Fig. 9d, e). Quantitative real-time PCR analysis confirmed a significant knockdown efficiency of ASO NEAT1 injected into the brain (Fig. S4). For the assessment of neurological deficits, a battery of well-defined neurological tests was used at 7 dpi (Fig. 9f–i). Mice exposed to MCAO displayed severe motor coordination deficits in the four tests analyzed. Treatment with ASO NEAT1, however, reversed the stroke-induced deficits on motor coordination at 7 dpi. Likewise, ASO NEAT1 yielded a significantly higher neuronal density at 7 dpi as assessed by histological analysis of the neuronal density (data not shown). These findings indicate that the knockdown of NEAT1 alleviates brain injury in MCAO mice both on the histological and even more relevant on the functional level.

## Discussion

The present study analyzed the role of the lncRNA NEAT1 and its underlying mechanisms under both in vitro and in vivo stroke conditions, thus establishing NEAT1 as a possible target for future stroke treatment. Previous work from other groups described a regulatory role of NEAT1 in settings of cerebral ischemia [17, 31, 32], albeit these studies predominantly remained descriptive. Lentiviral transfection with NEAT1 overexpression exacerbated cell death in MCAO rats and significantly aggravated neurological impairment after stroke in these studies [20]. On the contrary, downregulation of NEAT1 was found to protect neurons from apoptosis due to OGD/R in BV2 microglial cells by significantly inhibiting M1 polarization [21]. The present study observed a time-dependent upregulation of NEAT1 in hypoxic microglia and ischemic brain tissue, and silencing of NEAT1 directly promoted microglial cell viability, reduction of brain injury and neurological recovery in these experimental settings. Although the survival of both neurons and astrocytes is influenced by microglial responses per se, our data indicate that NEAT1 expression levels rather affect mutual communication processes between microglia and neurons than between microglia and astrocytes. Hence, knockdown of NEAT1 in microglia increases neuronal survival under hypoxic conditions in a co-culture system but has no impact on astrocyte survival under the very same conditions.

The precise mechanisms by which NEAT1 may interfere with the pathophysiology of stroke still remain elusive. However, in other diseases like hepatocellular carcinoma, previous work analyzing autophagy-related pathways suggested a role for NEAT1 in the triglyceride (TG) metabolism by being an upregulator of miR-372-3p in both RAPA-treated mice and in a cell model [33]. Further studies described a modulating role of NEAT1 with regard to the expression of adipose triglyceride lipase (ATGL) expression, and knockdown of NEAT1 attenuated human hepatocellular carcinoma cell growth through ATGL [34]. TG are the main contents of LD, whereas ATGL is the primary enzyme contributing to the breakdown of TG. Hence, we hypothesized whether or not NEAT1 affects lipid metabolism in the form of LD formation under in vitro or in vivo stroke conditions. In our study, the abundance of BODIPY^+^ microglia were significantly reduced after NEAT1 knockdown. Likewise, LD-related genes and proteins were also significantly downregulated after silencing of NEAT1 in microglia. These findings are consistent with some studies that suggest NEAT1 may control lipid uptake in macrophages. As such, Huang-Fu et al. used oxidized low-density lipoprotein to acquire lipid absorption in macrophage THP-1 cells. The detection outcomes showed a significant increase in NEAT1 expression relative to the normoxia group and further confirmed that low-level NEAT1-mediated paraspeckle formation suppresses lipid uptake [35]. In addition to this, small interfering RNA transfection of NEAT1 can significantly reduce total cholesterol, TG, and death of THP-1 macrophage cells [23]. Since microglia are the most well-characterized macrophage population residing in the central nervous system, these findings may also have imminent meaning for the central nervous system [36].

Focusing on primary microglia, the expression of LD in these cells was significantly upregulated in our OGD/R model. Consistent with our results, previous studies observed that neurodegenerative changes accompany significant agglomerations of LD in microglia [37]. Lin et al., for instance, described an increased number of LD containing microglia in a model of glucose-oxygen-serum deprivation. Such upregulation of LD was accompanied by an increased production of inflammatory cytokines. Inhibition of lipid formation significantly reduced both infarct size and motor function deficit in rats undergoing cerebral ischemia [38]. In our study, increased levels of LD were found seven days after stroke induction. The formation of LD under stroke conditions may be a consequence of different mechanisms that occur under such pathophysiological conditions, the first one being associated with rapid and widespread acute cell death. The latter results in the phagocytosis of dead cells by glial cells, which then may contribute to an overwhelming formation of LD upon stroke [39]. Another mechanism may involve the post-stroke disruption of the blood–brain barrier, allowing peripheral lipoprotein particles to enter the ischemic brain [40]. Other factors, including lipid transfer, oxidative stress, and nutrient deprivation may also have an impact in this scenario, albeit the specific mechanisms of LD agglomeration in microglia are still unclear. It is important to mention, however, that LD agglomeration is not only associated with pathology but also with physiological processes like aging. Indeed, aging results in the agglomeration of LD in residing microglia, displaying an increased activation of reactive oxygen species that is related to an excessive release of pro-inflammatory factors and impaired phagocytosis of microglia [11].

Although the precise mechanisms as to how LD formation is involved in the stroke pathology remain elusive, some information may be drawn from the known interplay between NEAT1 and autophagy. The latter is a critical degradation system for energy and cellular homeostasis, primarily as a defense mechanism that may prevent cell death in a stressful environment. Under stroke conditions, autophagy differentially affects stroke outcomes that can be either protective or harmful, depending on the activation state of autophagy [41]. Mounting evidence suggests a role for autophagy as part of the NEAT1 signaling pathway in various non-ischemic neurodegenerative disease models. As such, NEAT1 aggravates autophagy and cell injury in the MPTP-induced Parkinson's disease model [42, 43], whereas downregulation of NEAT1 inhibits Aβ uptake and degradation by regulating endocytosis-related gene expression in Alzheimer's disease [44]. Additional work established a connection between NEAT1 and autophagy, where NEAT1 regulates autophagy-related gene expression in glial cells, such as Atg3, Atg5, and Beclin1 [45]. In our preclinical stroke model, these markers were significantly increased, whereas silencing of NEAT1 significantly reduced the expression of these autophagy-related markers. Observing an increased level of transformation from LC3-I towards LC3-II in both hypoxic cells and stroke mice, indicates an increased formation of autophagosomes in our experiments. However, increased numbers of autophagosomes may not necessarily be a consequence of higher autophagy activity but also be due to either impaired fusion of autophagosomes and lysosomes or due to the degradation of autophagosomes [46]. Thus, we also detected the expression levels of p62, which serves as a cargo receptor for ubiquitinated substrates to autophagosomes for degradation. By detecting the expression of p62, elevated p62 reflects a decrease in the autophagic flux. Therefore, it is plausible that the combination of decreased LC3 and increased p62 could be reflecting an inhabitation of autophagosome degradation after knockdown of NEAT1.

Lipids are found in the cytoplasm of the intracellular compartment, which is also full of acid lipase containing lysosomes. Hence, autophagy signaling pathways also degrade LD. In a normal physiological state, autophagy is associated with phagocytosis, leading to the degradation of excess lipids and participating in a lipid metabolism called lipophagy (macrolipophagy or microlipophagy). Under nutrient deficiency conditions, lipids are encapsulated in a double membrane autophagosome and transported to lysosomes to be degraded into free fatty acids to provide energy for the body and maintain intracellular lipid homeostasis [47, 48]. Stimulating autophagy in our experimental paradigm by means of RAPA yielded an increased formation of intracellular LD formation, whereas 3-MA had the opposite effect. Knockdown of NEAT1 increased this effect even further, i.e., the formation of LD was reduced even further under such conditions. Such observations may be explained by two different mechanisms. Increased microglial activation and LD agglomeration have been associated with reduced phagocytosis [11]. Accordingly, NEAT1 knockdown may reduce microglial autophagy activation and thus contribute to impeding extracellular lipid uptake and clearance. Another mechanism is based on previous findings that demonstrated that Atg5 could target exogenous materials for endocytosis or phagocytosis via the LC3-dependent autophagy pathway [49, 50]. The autophagy-related gene Atg5, in turn, is known to be modulated by NEAT1 [45]. Thus, microglial NEAT1 silencing may impair endocytosis or phagocytosis of extracellular lipids through non-canonical autophagy.

Even though the current research work provides novel insight into the function of NEAT1 under stroke conditions, the administration of ASO NEAT1 before the onset of stroke may, of course, not entirely reflect the clinical stroke settings. Besides, due to the lower abundance of lncRNA, achieving a higher knockdown efficiency is necessary for studying the loss of lncRNA function in vivo compared to mRNA. Novel approaches like CRISPR-Cas9 lncRNA knockout may offer greater specificity and potentially fewer side effects in this context [17, 51]. Furthermore, our in vivo experiment has confirmed that knocking down NEAT1 can ameliorate post-stroke LD agglomeration and cortical cerebral blood flow. Elucidating a functionally relevant interaction between these two parameters, however, was beyond the scope of our study.

## Conclusion

The present study analyzed the role of NEAT1 under stroke conditions through a series of in vitro and in vivo experiments. Induction of OGD/R or stroke results in an increased expression of NEAT1, which is associated with enhanced LD formation and elevated expression levels of autophagy-related genes. Knockdown of NEAT1 in microglia reverses the aforementioned observations. Under knockdown conditions, stimulation of autophagy using RAPA reverses the downregulation of LD formation and PLIN2 expression patterns upon an OGD/R condition in microglia, whereas 3-MA promotes both downregulation of LD formation and PLIN2 expression. Regulating NEAT1 under hypoxic or ischemic brain conditions proved to be a vital signaling pathway, since knockdown of NEAT1 in mice not only reduced LD formation and autophagy activity but also resulted in better post-stroke neurological recovery. The present study therefore sheds new light into the complex signaling machine of NEAT1 under stroke conditions.

### Supplementary Information

Below is the link to the electronic supplementary material.

Supplementary file 1 (DOCX 2900 KB)

## Data Availability

The datasets generated and analyzed during the current study are available from the corresponding author on reasonable request.

## Abbreviations

BBB: Blood–brain barrier
DAMPs: Damage-associated molecular patterns
NEAT1: Nuclear paraspeckle assembly transcript 1
ASO: Antisense oligonucleotide
