# Supplementary material for: Knockdown of NEAT1 prevents post-stroke lipid droplet agglomeration in microglia by regulating autophagy
Source: Cell Mol Life Sci. 2024 Jan 12;81(1):30. doi: 10.1007/s00018-023-05045-7 (PMC10784396; doi:10.1007/s00018-023-05045-7)
Supplement: Supplementary file 1 — (DOCX 2900 KB) [file 18_2023_5045_MOESM1_ESM.docx]

**SUPPLEMENTARY MATERIALS**

**Knockdown of NEAT1 prevents post-stroke lipid droplet agglomeration in microglia by regulating autophagy**

**Yongli Pan^1^, Wenqiang Xin^1^, Wei Wei^1^, Lars Tatenhorst^1^, Irina Graf^1^, Aurel Popa-Wagner^2^, Stefan T Gerner^3^, Sabine E Huber^3^, Ertugrul Kilic^4^, Dirk M Hermann^2^, Mathias Bähr^1^, Hagen B Huttner^3^, and Thorsten R Doeppner^1,3,5-7^**

^1^University Medical Center Göttingen, Department of Neurology, Göttingen, Germany

^2^Department of Neurology, University Hospital Essen, University of Duisburg-Essen,

Essen, Germany

^3^Department of Neurology, University of Giessen Medical School, Giessen, Germany

^4^Department of Physiology, Istanbul Medeniyet University, Faculty of Medicine, Istanbul, Turkey

^5^Department of Anatomy and Cell Biology, Medical University of Varna, Varna, Bulgaria

^6^Center for Mind, Brain and Behavior (CMBB), University of Marburg and Justus Liebig University Giessen, Germany

^7^Research Institute for Health Sciences and Technologies (SABITA), Medipol University, Istanbul, Turkey

**Correspondence:**

Thorsten R. Doeppner, MD-M.Sc.

Department of Neurology

University of Giessen Medical School, Giessen, Germany

Phone: +49-641- 98545393

Email: [thorsten.doeppner@neuro.med.uni-giessen.de](mailto:thorsten.doeppner@neuro.med.uni-giessen.de)

**MATERIALS AND METHODS S1**

**Neurobehavioral tests**

All mice were trained 1-2 days before induction of stroke to ensure accurate test performance. Post-stroke neurological recovery was assessed using a battery of four motor coordination tests, i.e., the rotarod, tightrope, balance beam, and paw slip test [1]. The rotarod was carried out to assess locomotor function, in which speed increases linearly from 4 to 30 rpm. The time until each mouse dropped was measured, with a maximal testing time of 300 s [2], whereas in the tightrope test, the time until reaching one of the platforms was registered and statistically analyzed (maximal testing time 60 s). The tightrope test results were evaluated employing a validated score ranging from 0 to 20. The details of the tightrope test score sheet can be found in **Table S4**. The balance beam test was used to measure asymmetrical coordination. The beam was placed up at a height of 50 cm. The time the animal needed to reach the platform (maximal testing time 60 s) and the number of paw slips were recorded [3]. Data are given as the so-called calculated laterality index.

**References**

1. Doeppner, T., et al., *Ischemic Post-Conditioning Induces Post-Stroke Neuroprotection via Hsp70-Mediated Proteasome Inhibition and Facilitates Neural Progenitor Cell Transplantation.* Molecular neurobiology, 2017. **54**(8): p. 6061-6073.

2. Silva de Carvalho, T., et al., *Post-ischemic protein restriction induces sustained neuroprotection, neurological recovery, brain remodeling, and gut microbiota rebalancing.* Brain, behavior, and immunity, 2022. **100**: p. 134-144.

3. Hermann, D., et al., *Sustained neurological recovery induced by resveratrol is associated with angioneurogenesis rather than neuroprotection after focal cerebral ischemia.* Neurobiology of disease, 2015. **83**: p. 16-25.

**SUPPLEMENTARY FIGURES AND TABLES**

**
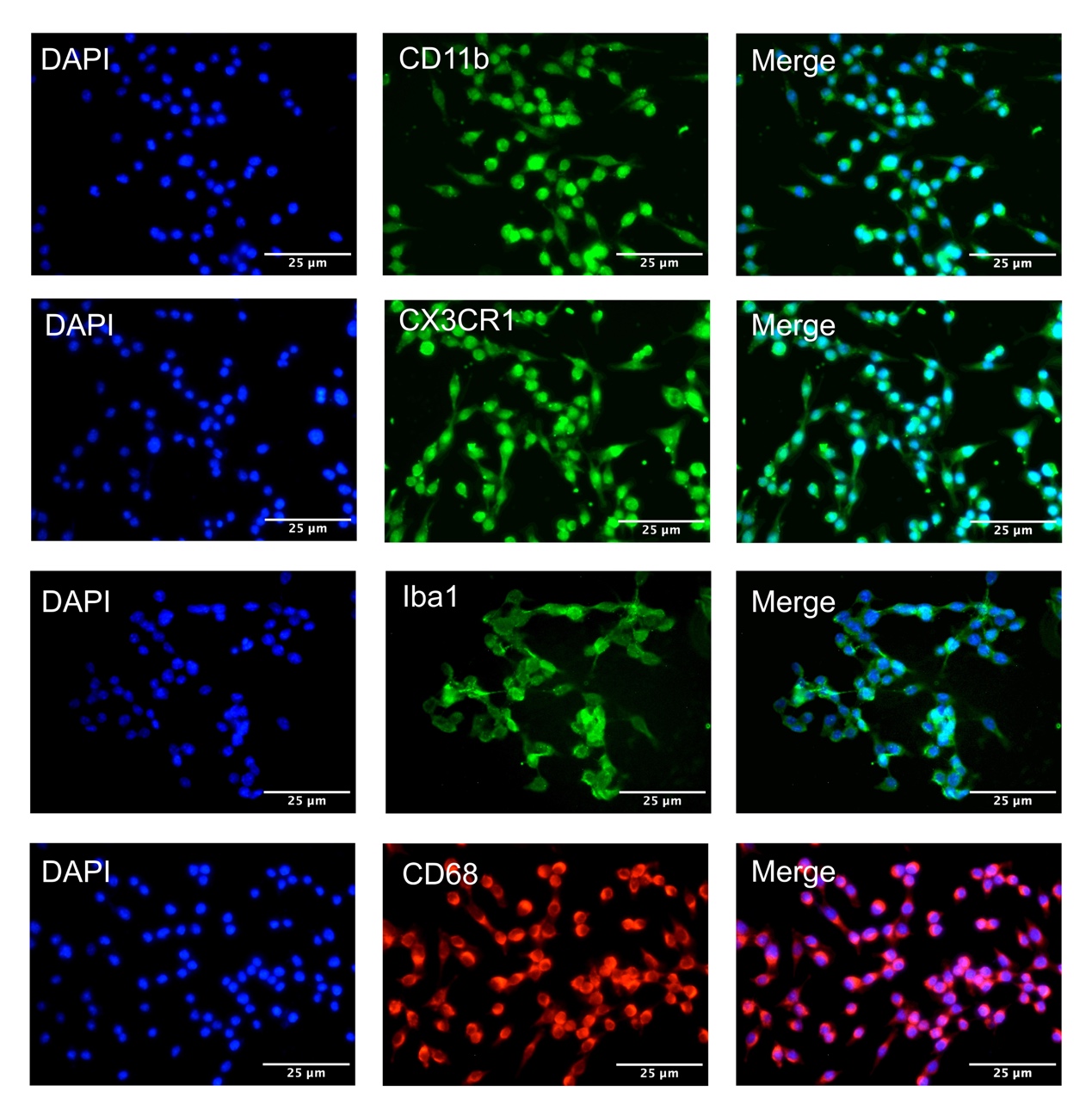
**

**Figure S1. Characterization of primary microglia.** Primary microglia were isolated from cerebral cortices and hippocampi of newborn WT C57BL/6J mice. Immunofluorescence images labeling CD11b, CX3CR1, and Iba1 in green and CD68 in red under normoxia conditions, while the nuclei were counterstained with DAPI in blue (top to bottom).

**
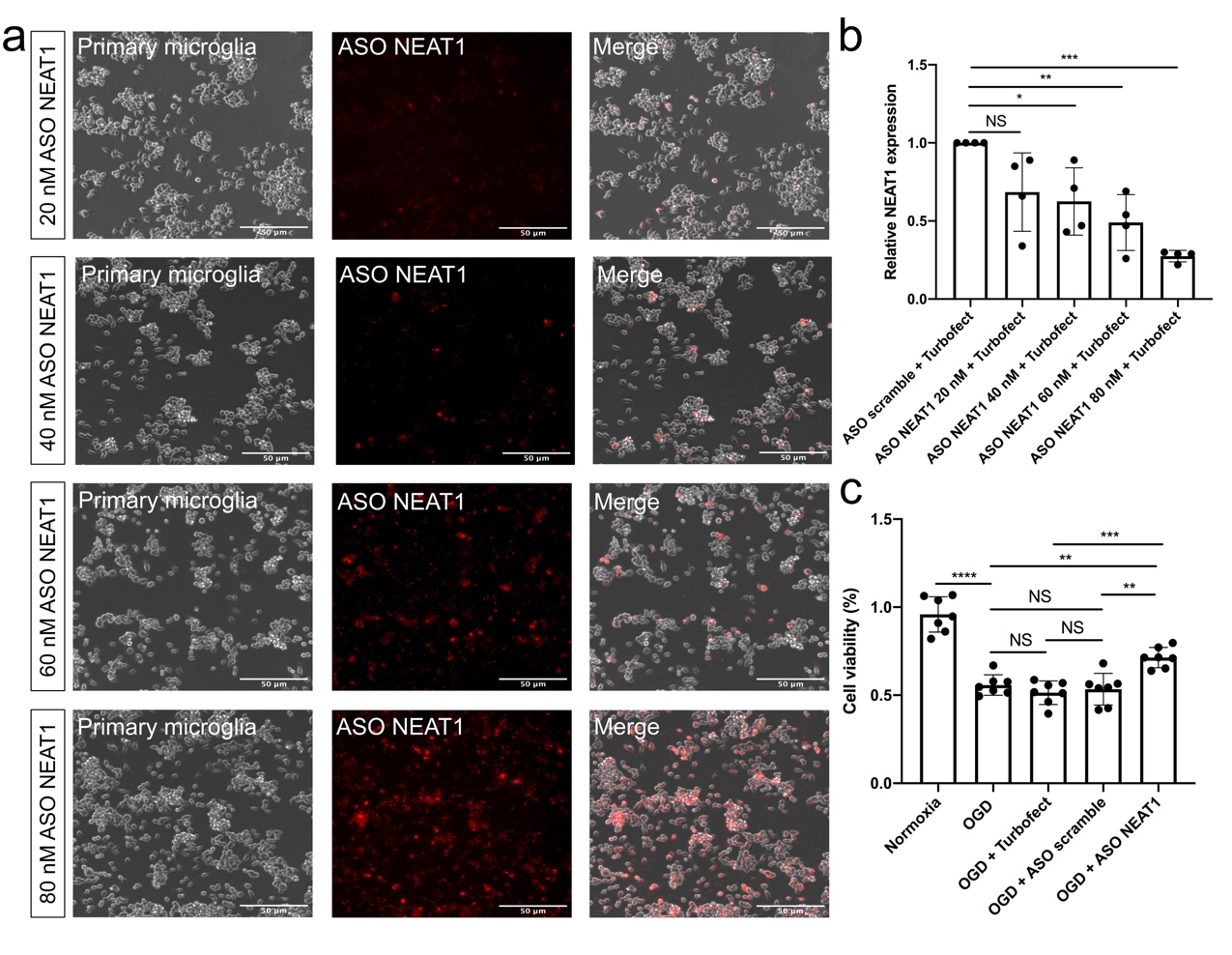
**

**Figure S2. Knockdown of NEAT1 promoted cell survival of primary microglia exposed to hypoxia. (a)** Bright-field microscopy images of microglia transfected with ASO NEAT1 at different concentrations. (**b)** qRT-PCR validation of the transfection efficiency of ASO NEAT1 in comparison to PPIA. (**c)** MTT assay analysis of cell viabilities of microglia treated with ASO NEAT1. All results are expressed as mean ± standard deviation and analyzed by one-way ANOVA followed by Tukey's post-hoc-test. NS, no significance, * *p* <0.05, ** *p* <0.01, *** *p* <0.001, and **** *p* <0.0001. Abbreviations: NEAT1, nuclear paraspeckle assembly transcript 1; ASO, antisense oligonucleotide; OGD, oxygen-glucose-deprivation; PPIA, peptidylprolyl isomerase A.


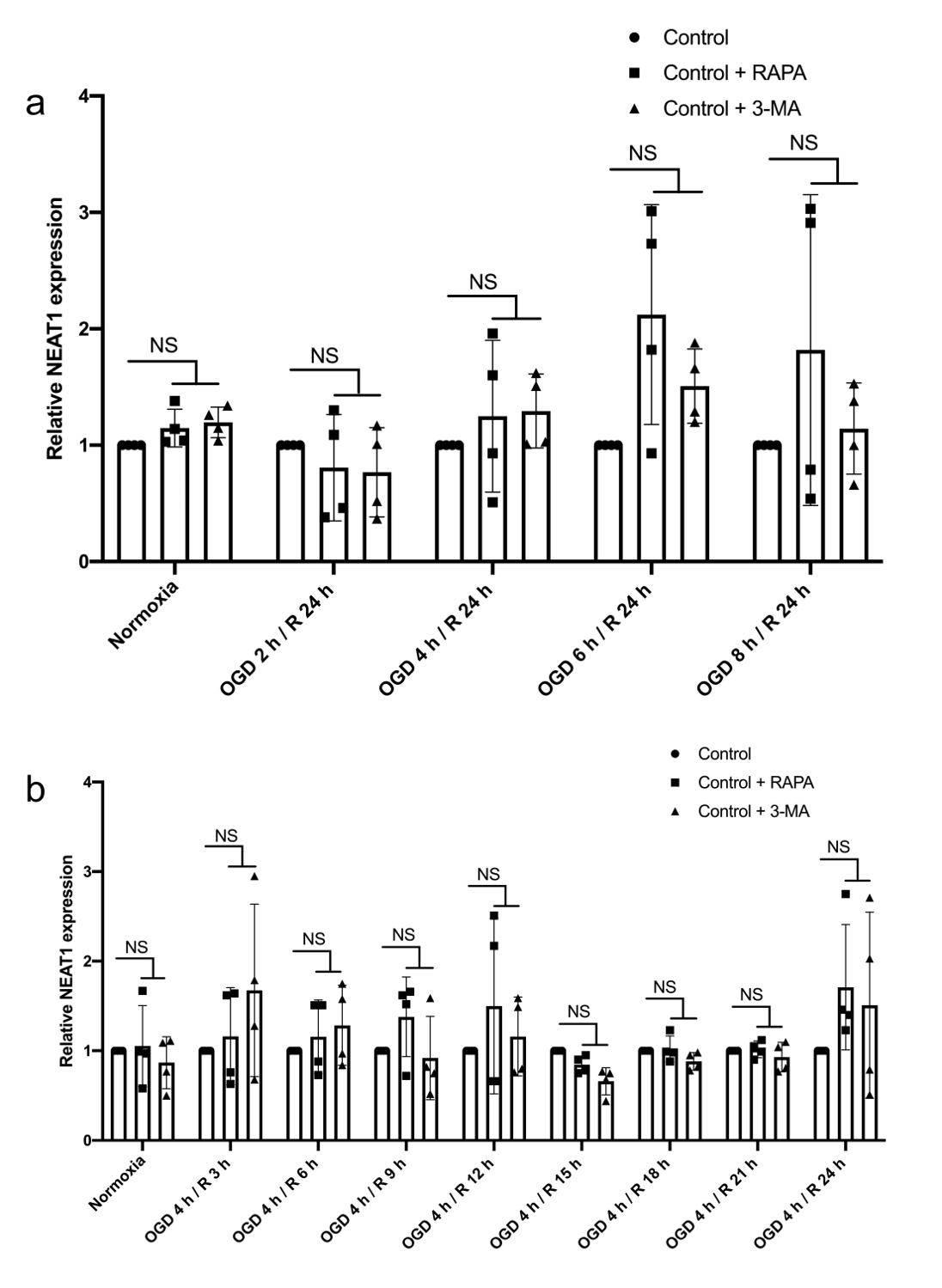


**Figure S3. Regulation of autophagy does not affect NEAT1 expression in primary microglia under normoxic or hypoxic conditions.** Microglial cells were treated with RAPA or 3-MA under normoxic or hypoxic conditions followed by qRT-PCR in comparison to PPIA. **(a)** Corresponding NEAT1 expression at different time points of OGD. **(b)** The corresponding NEAT1 expression at different time points of reoxygenation. All results are expressed as mean ± standard deviation and analyzed by one-way ANOVA followed by Tukey's post-hoc-test. NS, no significance. Abbreviations: NEAT1, nuclear paraspeckle assembly transcript 1; PPIA, peptidylprolyl isomerase A; OGD/R, oxygen-glucose deprivation/reoxygenation; 3-MA, 3-methyladenine; RAPA, rapamycin.

**
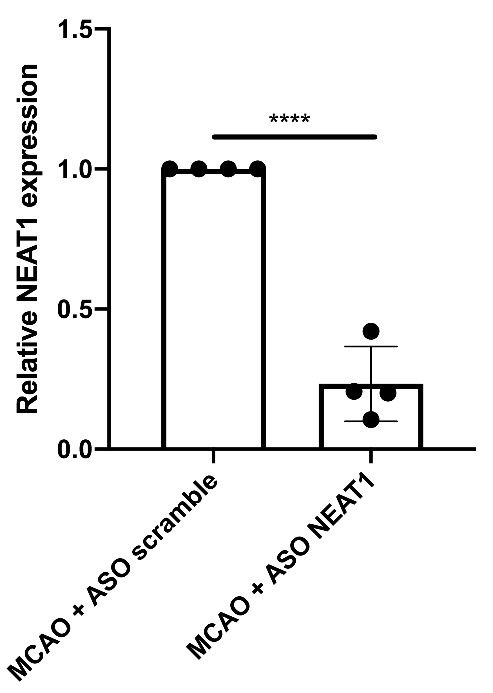
**

**Figure S4.** **Knockdown efficiency determined by qRT-PCR in mice**. qRT-PCR validation of the transfection efficiency of ASO NEAT1 in comparison to PPIA. All results are expressed as mean ± standard deviation and Student's *t*-test (n = 4 mice per experimental group) was used to compare between groups. **** *p* <0.0001. Abbreviations: NEAT1, nuclear paraspeckle assembly transcript 1; ASO, antisense oligonucleotide; PPIA, peptidylprolyl isomerase A; MCAO, middle cerebral artery occlusion.

**Table S1. Experimental groups and survival rates of mice.**

| WB analysis, qRT-PCR and immunofluorescence | | | | Behavior tests | | |
| --- | --- | --- | --- | --- | --- | --- |
| Groups | **Total** | **Dead** | **Survival** | **Total** | **Dead** | **Survival** |
| Sham | 8 | 0 | 8 |  |  |  |
| MCAO 1 dpi | 8 | 0 | 8 |  |  |  |
| MCAO 3 dpi | 6 | 1 | 5 |  |  |  |
| MCAO 5 dpi | 5 | 0 | 5 |  |  |  |
| MCAO 7 dpi | 10 | 2 | 8 |  |  |  |
| MCAO 14 dpi | 4 | 1 | 3 |  |  |  |
| Sham + ASO scramble | 12 | 0 | 12 | 12 | 0 | 12 |
| Sham + ASO NEAT1 | 13 | 0 | 13 | 12 | 0 | 12 |
| MCAO + ASO scramble | 16 | 4 | 12 | 12 | 0 | 12 |
| MCAO + ASO NEAT1 | 18 | 6 | 12 | 12 | 0 | 12 |

A total of 41 mice was used for sham and MCAO groups in order to detect the post-stroke expression of NEAT1 and the trend of lipid change. Four animals out of these 41 died at different time points (3 dpi, 7 dpi, and 14 dpi). For the remainder of the study, 59 mice were used in the following *in* *vivo* experiments including western blots, qRT-PCR assays, immunofluorescence staining and behavior tests. Of these 59 mice, 10 animals died or had to be sacrificed before reaching the end of the experiment. Finally, a total 48 mice were used for the behavioral test analyses. None of these animals died. Abbreviations: dpi, day post-ischemia; MCAO, middle cerebral artery occlusion; NEAT1, nuclear paraspeckle assembly transcript 1; ASO, antisense oligonucleotide.

**Table S2.** **Sequence information of qRT-PCR analysis primers.**

| Gene name | Sequence (5′ - 3′) |
| --- | --- |
| PPIA F | GAGCTGTTTGCAGACAAAGTTC |
| PPIA R | CCCTGGCACATGAATCCTGG |
| NEAT1 F | GTAATTTTCGCTCGGCCTGG |
| NEAT1 R | TACCCGAGACTACTTCCCCA |
| TREM2 F | CCCACCTGGCTGTTGTCCTT |
| TREM2 R | TCGCTACCGTGGAGGCTCTG |
| PLIN2 F | ACACCCTCCTGTCCAACATC |
| PLIN2 R | AAGGGACCTACCAGCCAGTT |
| Atg3 F | ACACGGTGAAGGGAAAGGC |
| Atg3 R | TGGTGGACTAAGTGATCTCCAG |
| Atg5 F | TGTGCTTCGAGATGTGTGGTT |
| Atg5 R | ACCAACGTCAAATAGCTGACTC |
| Beclin1 F | ATGGAGGGGTCTAAGGCGTC |
| Beclin1 R | TGGGCTGTGGTAAGTAATGGA |
| STAT3 F | CTTGTCTACCTCTACCCCGACAT |
| STAT3 R | GATCCATGTCAAACGTGAGCG |

**Table S3. Antibodies information for western blots.**

| **Antibody** | **Concentration** | **Supplier** | **Cat. No.** | **Species** |
| --- | --- | --- | --- | --- |
| Anti-TREM2 | 1:1,000 | ThermoFisher, USA | PA5-87933 | rabbit |
| Anti-PLIN2 | 1:1,000 | ProGen, Germany | GP42 | guinea pig |
| Anti-LC3 | 1:1,000 | Abcam, UK | ab128025 | rabbit |
| Anti-p62 | 1:1,000 | Abcam, UK | ab109012 | rabbit |
| Anti-Tublin | 1:10,000 | GeneTex, USA | GTX628802 | mouse |
| Anti-β-actin | 1:10,000 | Abcam, UK | ab6276 | mouse |
| Anti-GAPDH | 1:10,000 | GeneTex, USA | GTX627408 | mouse |
| Anti-rabbit | 1:10,000 | Abcam, UK | ab97051 | goat |
| Anti-mouse | 1:10,000 | Abcam, UK | ab97023 | goat |
| Anti-guinea pig | 1:10,000 | Abcam, UK | ab6908 | goat |
| Anti-chicken | 1:10,000 | Santa Cruz Biotechnology | sc-2428 | goat |
|  |  |  |  |  |

**Table S4.** **Tightrope test score sheet.**

| **Score** | **Time (s)** | **Platform arrival** |
| --- | --- | --- |
| 20 | 1-6 | + |
| 19 | 7-12 | + |
| 18 | 13-18 | + |
| 17 | 19-24 | + |
| 16 | 25-30 | + |
| 15 | 31-36 | + |
| 14 | 37-42 | + |
| 13 | 43-48 | + |
| 12 | 49-54 | + |
| 11 | 55-60 | + |
| 10 | 55-60 | − |
| 9 | 49-54 | − |
| 8 | 43-48 | − |
| 7 | 37-42 | − |
| 6 | 31-36 | − |
| 5 | 25-30 | − |
| 4 | 19-24 | − |
| 3 | 13-18 | − |
| 2 | 7-12 | − |
| 1 | 1-6 | − |
| 0 | 0 | − |

The tightrope test was carried out three times per day, and the mean values were recorded. The results were assessed according to both time on the rope (in seconds) and platform arrival (“+” for arrival and “−” for non-arrival). The scores ranged from 0 (minimum) to 20 (maximum).

**Supplementary negative staining controls in primary cells and in mice**

**
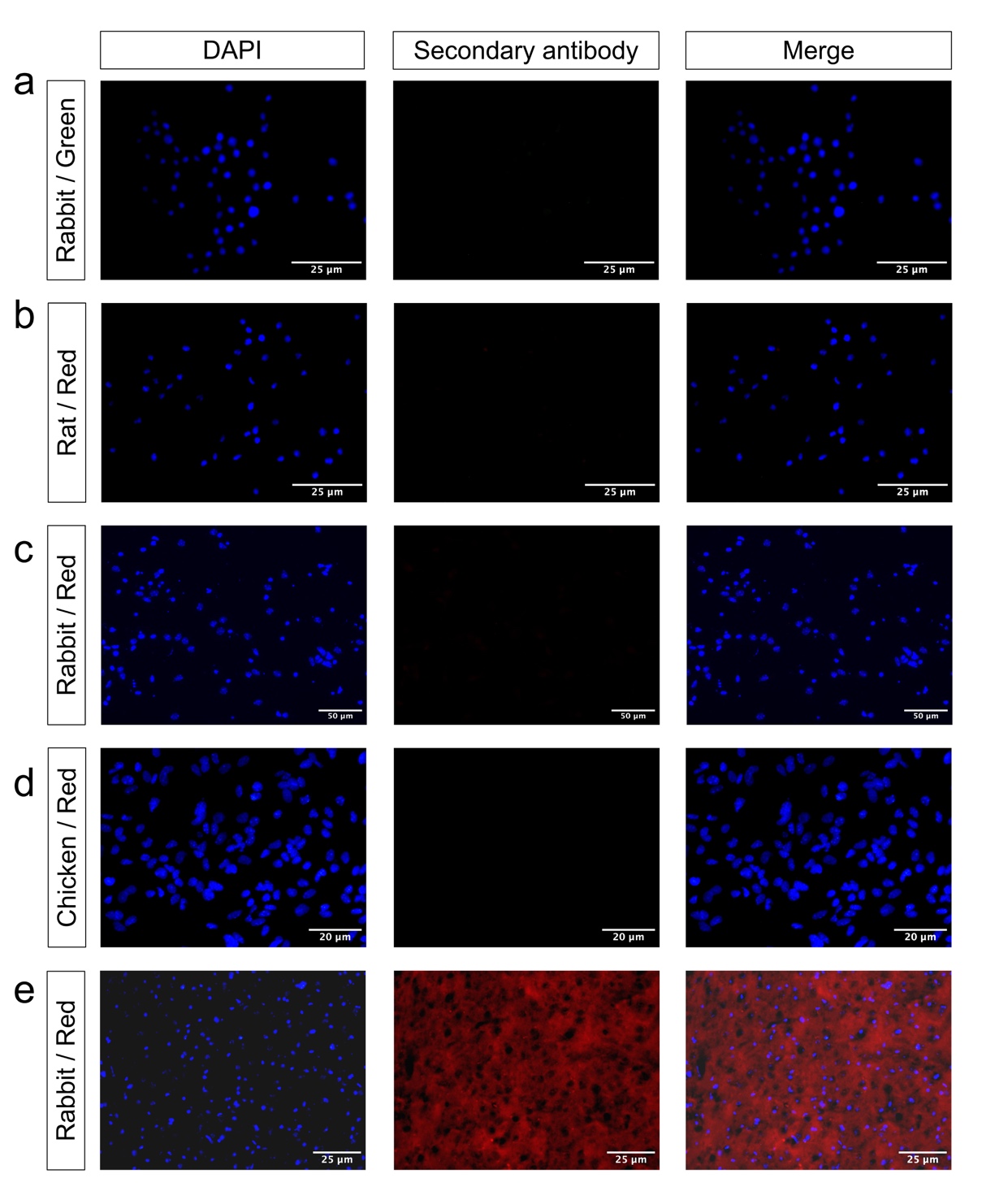
**

**(a)** As a negative control, the secondary antibody Alexa 488 donkey anti-rabbit IgG (1:250, green) was incubated with primary microglia, which does not yield any unspecific staining in cells. **(b)** As a negative control, the secondary antibody Cy3 donkey anti-rat IgG (1:250, red) was incubated with primary microglia, showing no unspecific staining on the slides. **(c)** As a negative control, the secondary antibody Cy3 donkey anti-rabbit IgG (1:250, green) was incubated with primary neurons, which does not yield any unspecific staining in cells. **(d)** As a negative control, the secondary antibody Cy3 donkey anti-rabbit IgG (1:250, red) was incubated with primary astrocytes, showing no unspecific staining on the slides. **(e)** As a negative control, the secondary antibody Cy3 donkey anti-rabbit IgG (1:250, red) was incubated with tissue slides, no unspecific staining was observed in this section.

**Supplementary full scans of western blots.**


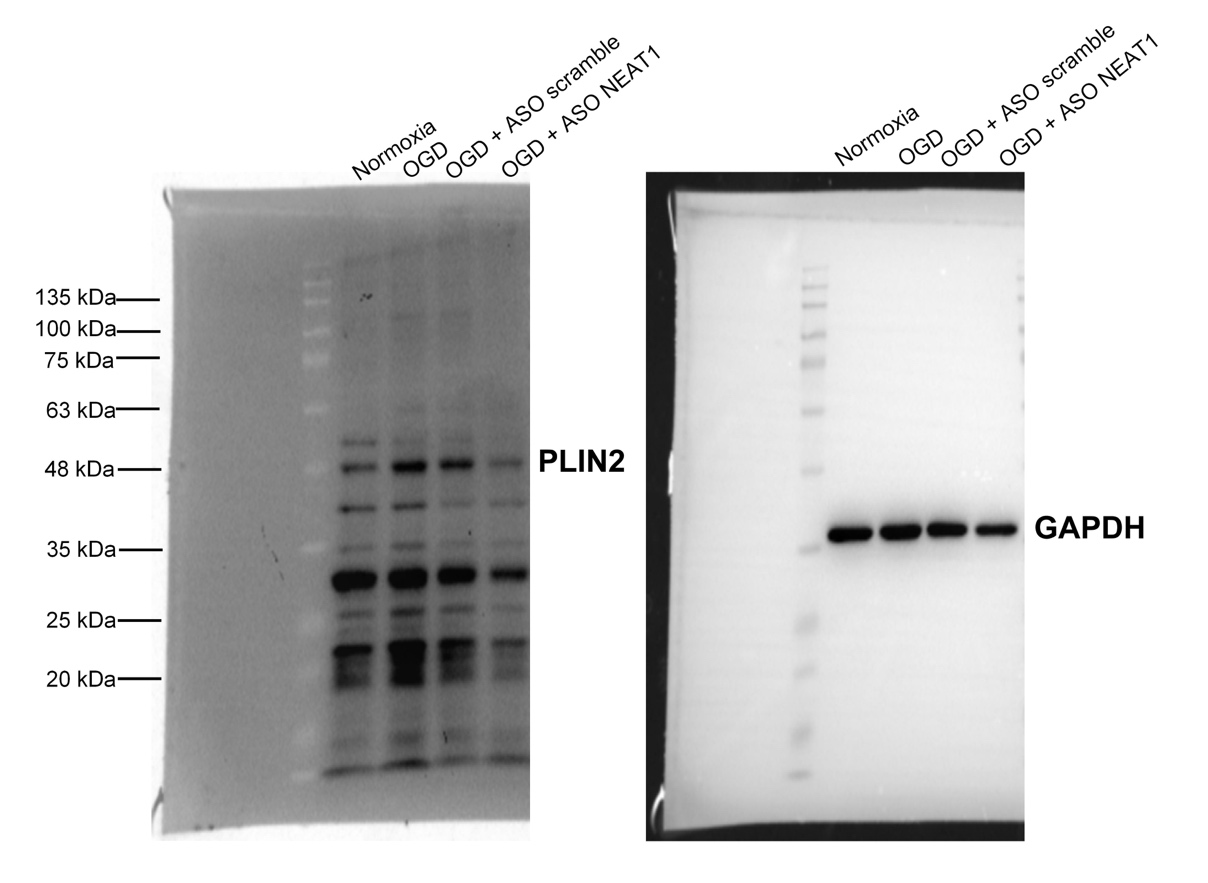


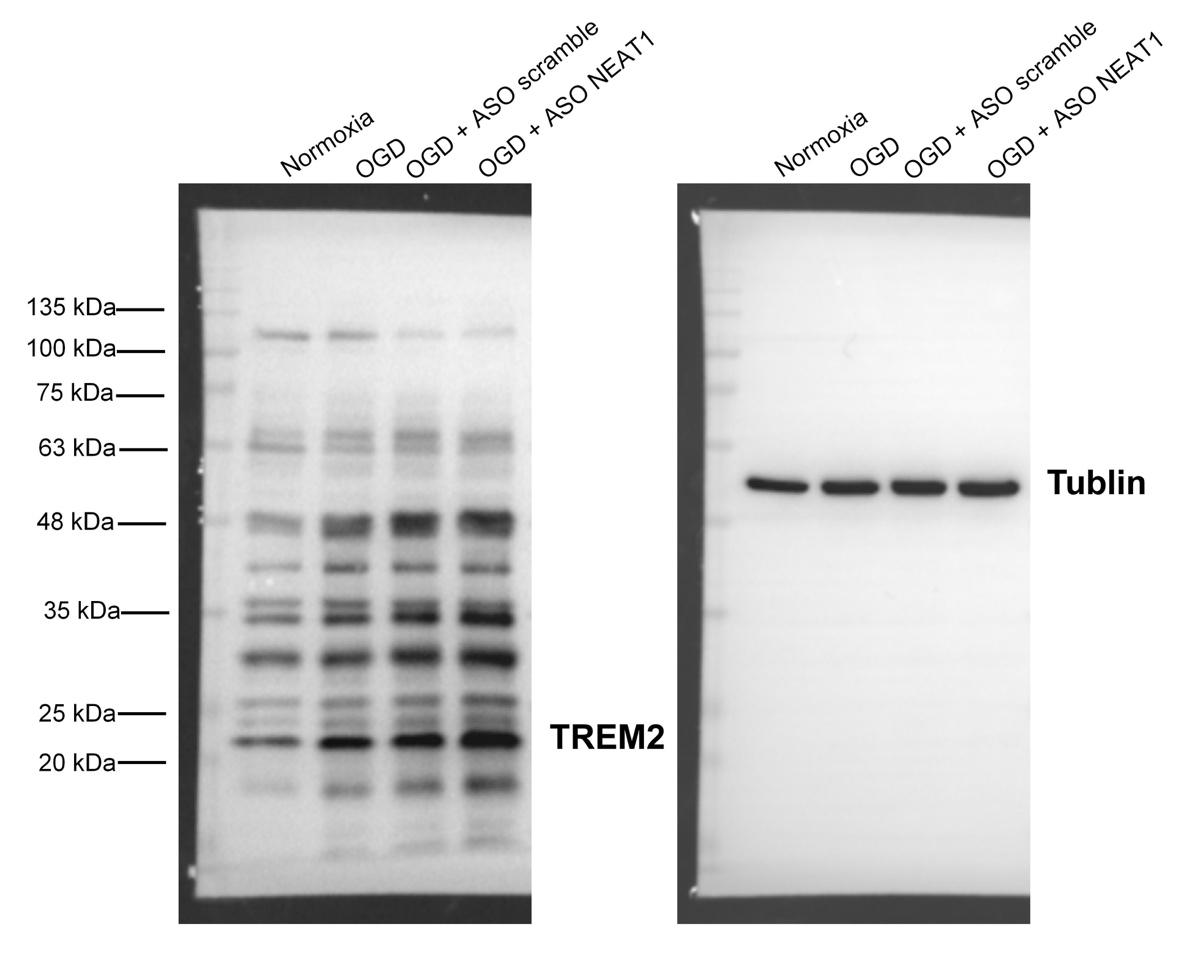


**Full scans of western blots are shown in Figure 2e.** PLIN2 and GAPDH, TREM2 and Tublin.

**
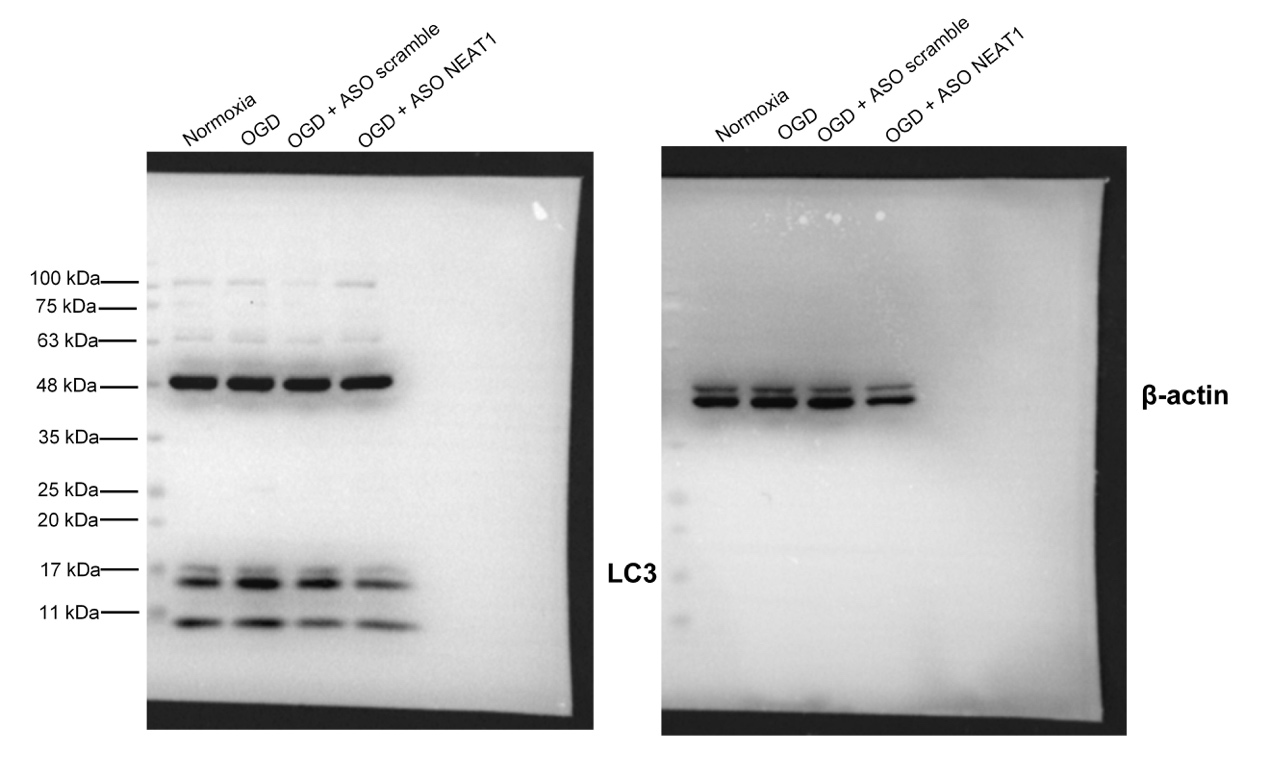
**

**Full scans of western blots are shown in Figure 3e.** LC3 and β-actin.

**
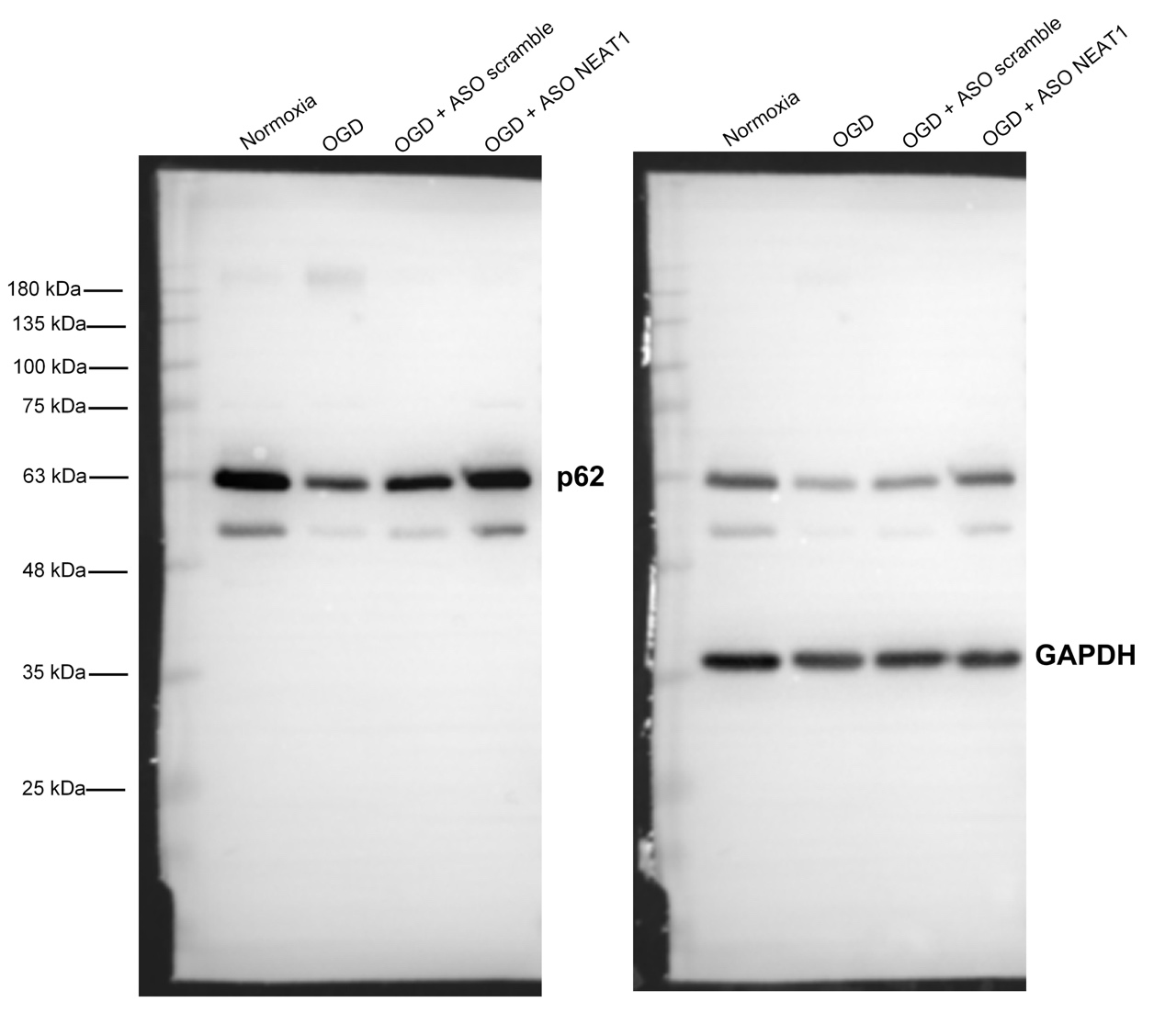
Full scans of western blots are shown in Figure 3f.** p62 and GAPDH.


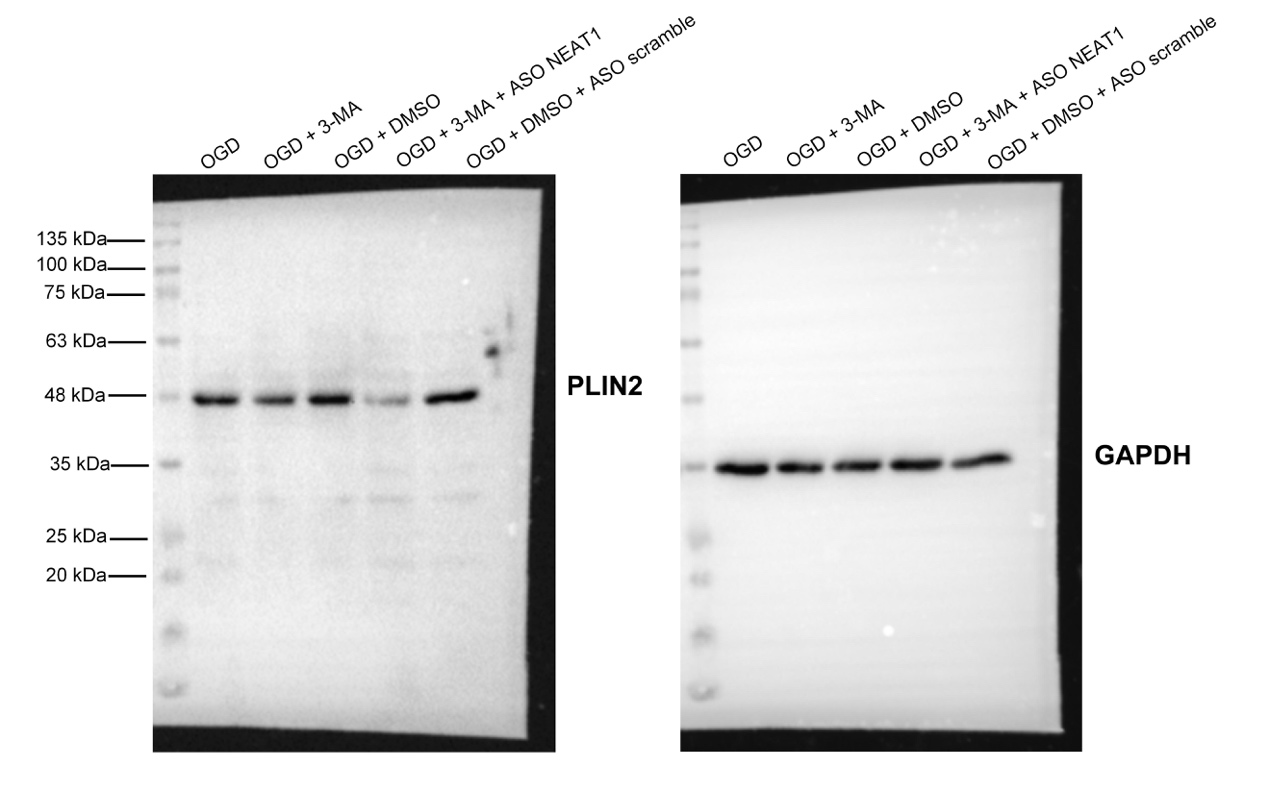


**Full scans of western blots are shown in Figure 4e.** PLIN2 and GAPDH.


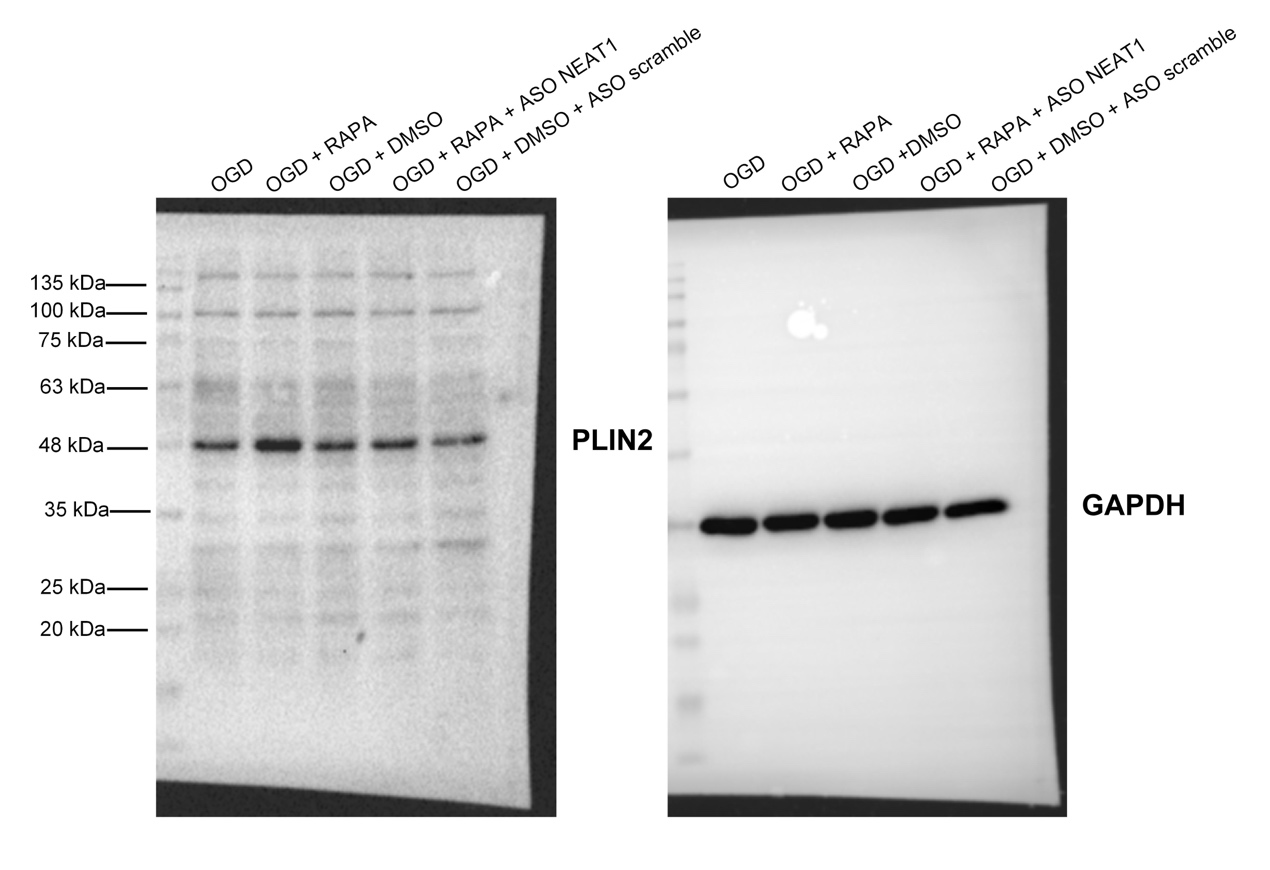


**Full scans of western blots are shown in Figure 4f.** PLIN2 and GAPDH.

**
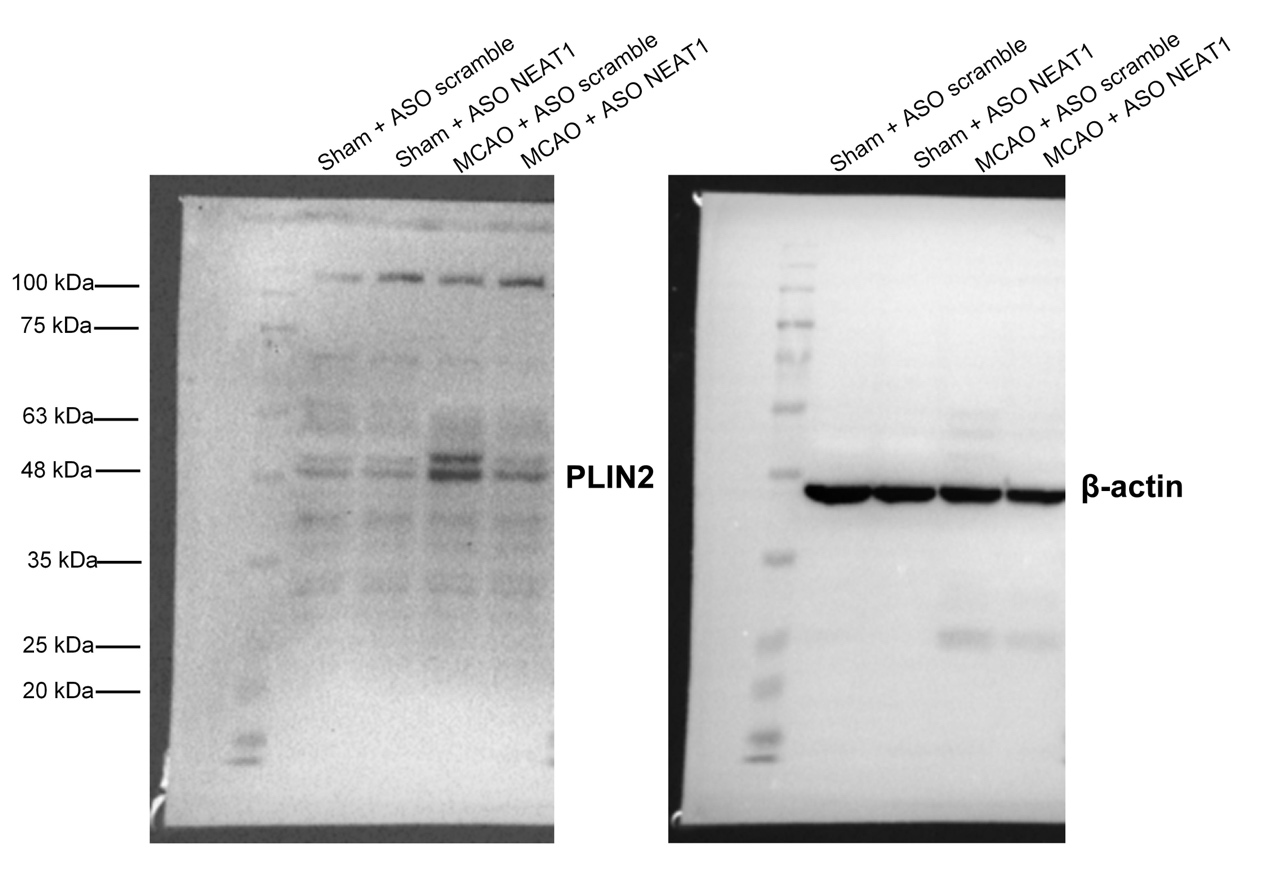
Full scans of western blots are shown in Figure 8c.** PLIN2 and β-actin.


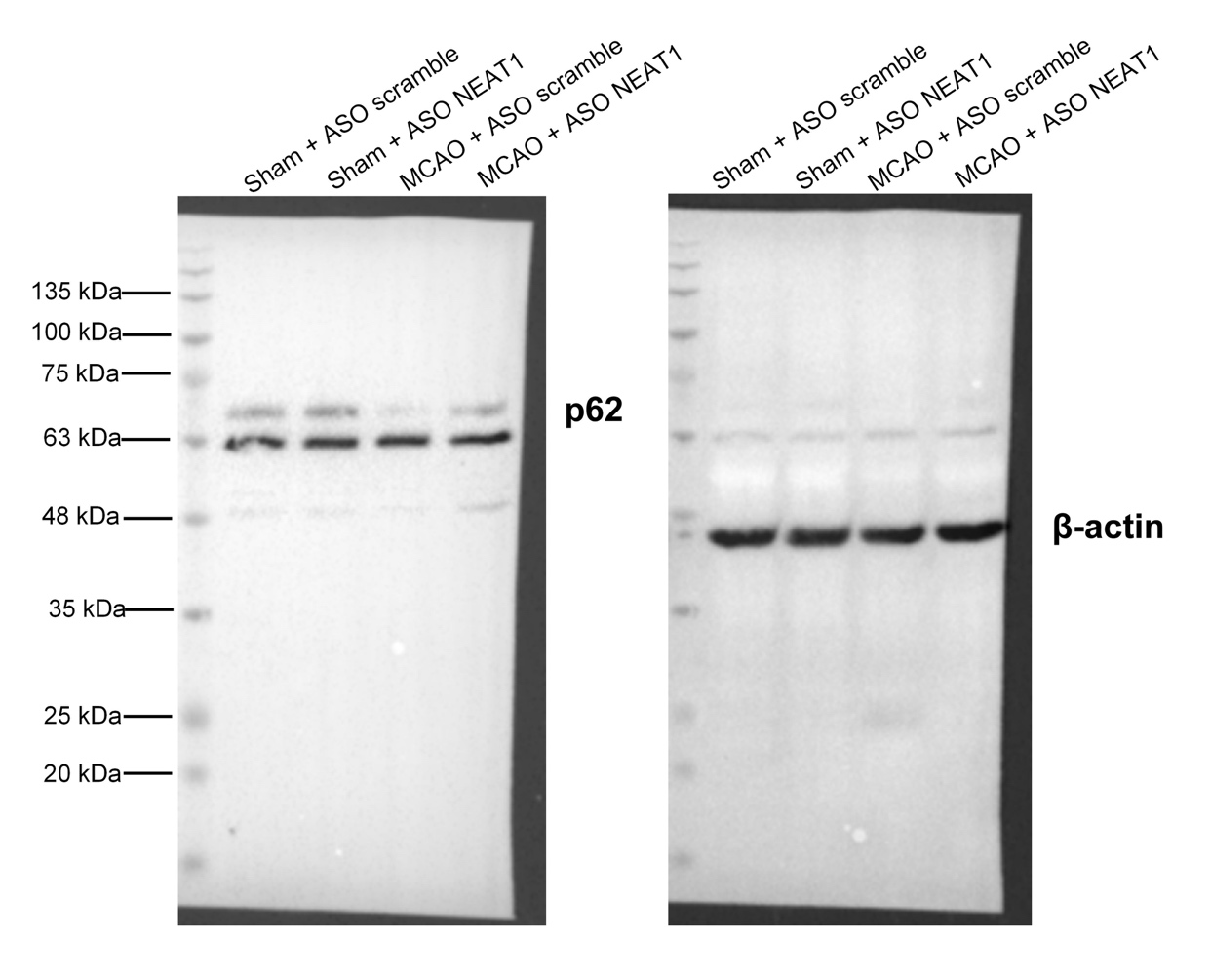


**Full scans of western blots are shown in Figure 8g.** p62 and β-actin.

**
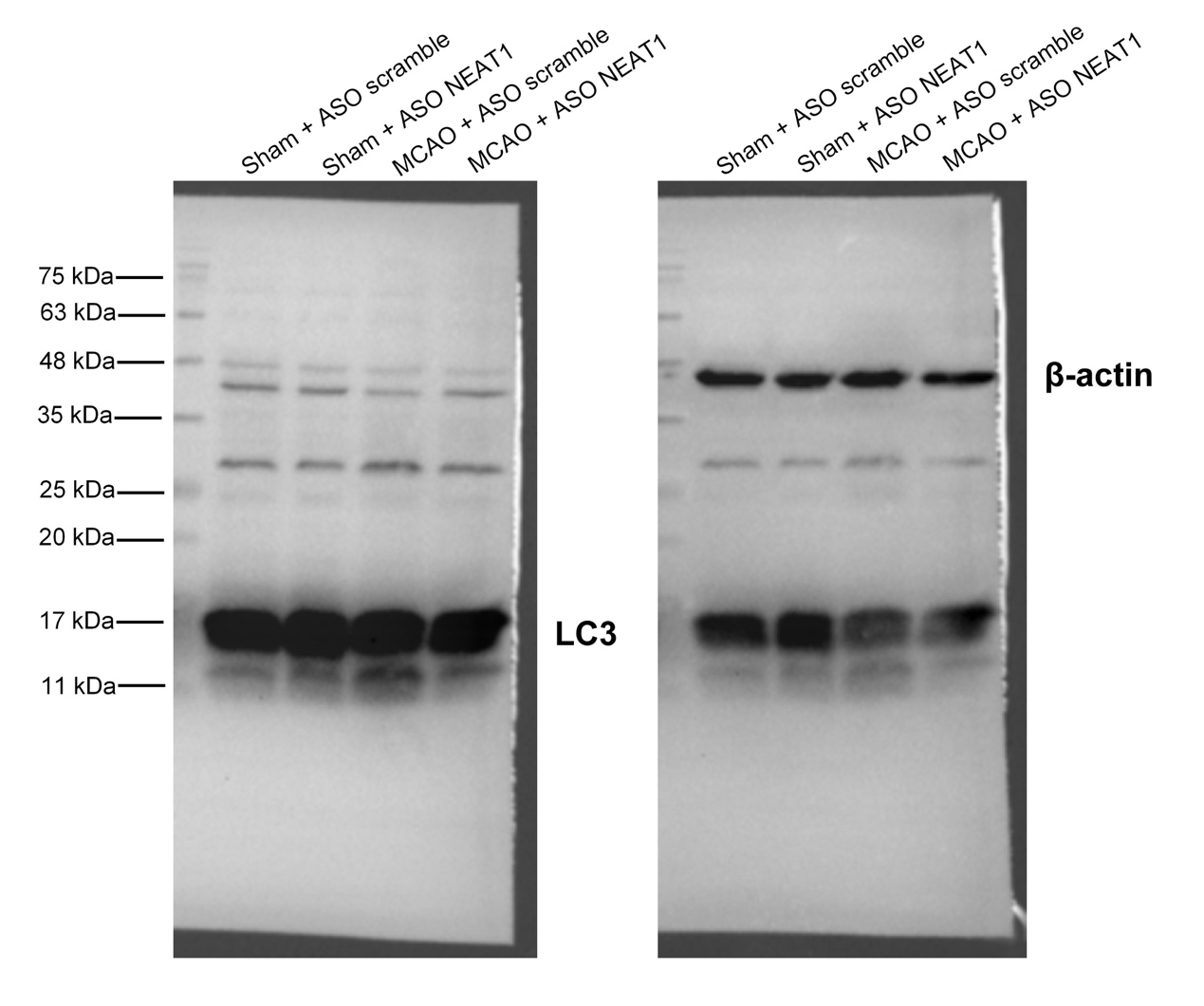
Full scans of western blots are shown in Figure 8i.** LC3 and β-actin.
